# Regulation of immunological tolerance by the p53-inhibitor iASPP

**DOI:** 10.1038/s41419-023-05567-9

**Published:** 2023-02-06

**Authors:** Elliot H. Akama-Garren, Paul Miller, Thomas M. Carroll, Michael Tellier, Gopinath Sutendra, Ludovico Buti, Justyna Zaborowska, Robert D. Goldin, Elizabeth Slee, Francis G. Szele, Shona Murphy, Xin Lu

**Affiliations:** 1 Ludwig Institute for Cancer Research, Nuffield Department of Clinical Medicine, University of Oxford, Oxford, OX3 7DQ UK; 2 Harvard-MIT Health Sciences and Technology, Harvard Medical School, Boston, MA 02115 USA; 3 Sir William Dunn School of Pathology, University of Oxford, Oxford, OX1 3RE UK; 4 Department of Medicine, University of Alberta, Edmonton, AB T6G 2B7 Canada; 5 Charles River Laboratories, Leiden, Netherlands; 6 Centre for Pathology, St. Mary’s Hospital, Imperial College, London, W2 1NY UK; 7 Department of Physiology, Anatomy and Genetics, University of Oxford, Oxford, OX1 3PT UK

**Keywords:** Cell death and immune response, Tumour immunology, Autoimmunity, Tumour-suppressor proteins

## Abstract

Maintenance of immunological homeostasis between tolerance and autoimmunity is essential for the prevention of human diseases ranging from autoimmune disease to cancer. Accumulating evidence suggests that p53 can mitigate phagocytosis-induced adjuvanticity thereby promoting immunological tolerance following programmed cell death. Here we identify Inhibitor of Apoptosis Stimulating p53 Protein (iASPP), a negative regulator of p53 transcriptional activity, as a regulator of immunological tolerance. iASPP-deficiency promoted lung adenocarcinoma and pancreatic cancer tumorigenesis, while iASPP-deficient mice were less susceptible to autoimmune disease. Immune responses to iASPP-deficient tumors exhibited hallmarks of immunosuppression, including activated regulatory T cells and exhausted CD8^+^ T cells. Interestingly, iASPP-deficient tumor cells and tumor-infiltrating myeloid cells, CD4^+^, and γδ T cells expressed elevated levels of PD-1H, a recently identified transcriptional target of p53 that promotes tolerogenic phagocytosis. Identification of an iASPP/p53 axis of immune homeostasis provides a therapeutic opportunity for both autoimmune disease and cancer.

## Introduction

One of the most fundamental functions of the immune system is the clearance of dead cells following normal tissue turnover, malignancy, injury, or infection. In this capacity, the immune system exhibits remarkable capability to discriminate whether to respond in an immunogenic or tolerogenic manner depending on the nature of cell death [1]. The daily homeostatic turnover of billions of cells rarely invokes an immune response, while the corpses of relatively few infected or malignant cells can elicit robust antigen-specific immune responses and immunological memory. Defects in this dichotomy can lead to human disease. For example, mistaken immunogenic phagocytosis in combination with epitope spreading contributes to autoimmune disease, while tumors can evade the immune system by promoting tolerogenic phagocytosis [2, 3]. Fundamentally, adaptive immunogenicity requires simultaneous antigenicity and adjuvanticity, and although cell death and subsequent phagocytosis almost universally promote antigen presentation, cell death can elicit varying adjuvanticity.

The nature of the immune response to cell death, whether immunogenic or tolerogenic, is relayed from the innate to adaptive immune system by manipulation of co-stimulation and cytokine-mediated adjuvanticity following phagocytosis [4]. Apoptosis or programmed cell death is typically immunologically silent while necrosis stimulates inflammation [5]. For example, phagocytosis of apoptotic cells can generate anergic cytotoxic T lymphocytes due to insufficient co-stimulation, stimulate immune checkpoint pathways, promote immunosuppressive cytokine release, and induce systemic antigenic tolerance [6–9]. In contrast, inflammatory cell death such as necrosis, pyroptosis, or ferroptosis can induce inflammation via recognition of pathogen-associated molecular patterns, damage-associated molecular patterns (DAMPs), inflammasome activation, release of pro-inflammatory cytokines, and licensing CD4^+^ T cell help of cytotoxic T cell development [1, 10–12]. The tissue context of the responding antigen-presenting cell (APC) might also influence the immunological consequences of cell death, as mature MHCII^hi^CD80^hi^ dendritic cells (DCs) are necessary to mount effective immune responses [13], while immature DCs are tolerogenic [14]. Ferroptosis of tumor neutrophil myeloid-derived suppressor cells can be immunosuppressive and promote tumor growth in vivo [15]. Therefore, the identity, location, activation status, and life history of the dying cell as well as those of the cell engulfing it all influence the nature of the immune response to cell death.

Physiologic apoptosis and unnatural cell death elicit fundamentally different immune responses, yet the pathways intrinsic to dying cells responsible for modulating immune homeostasis and phagocytic response remain poorly characterized. However, master regulators of cell death, such as p53, may play key roles in the establishment of an inherently immune activating or tolerizing transcriptional program [16, 17]. P53 potently induces apoptosis or cellular senescence following genotoxic stress by transactivating and trans-repressing hundreds of target genes, including genes involved in immune regulation [18–20]. Dominant negative and missense mutations in *TP53* or its effectors Bim, p21, Gadd45a, and Fas are associated with an increasing number of autoimmune diseases [21–32], demonstrating the importance of p53 in maintaining immune tolerance. Expression of the p53 target Fas by apoptotic cells and subsequent Fas-Fas ligand (FasL) signaling in responding lymphocytes is necessary for APC release of the immunosuppressive cytokines TGF-β and IL-10, and maintenance of immune privilege and immune tolerance [33–38]. More recent studies have identified novel roles of p53 in regulation of both innate and adaptive immune responses with potential non-cell-autonomous influences on tumor immunology [39, 40], including upregulation of PD-L1 and PD-1H [17, 41] and suppression of pro-inflammatory cytokine secretion [42, 43]. Although recent evidence suggests that dying cells communicate with the immune system via p53, the mechanisms by which p53 and its regulators influence immunological tolerance remain largely unknown.

One of the most ancient regulators of p53 is the Ankyrin repeat domain, SH3 domain and Proline rich sequence containing Protein (ASPP) family of proteins [44]. Inhibitor of Apoptosis Stimulating p53 Protein (iASPP) is an ASPP family member that binds and inhibits the transcriptional activity of p53 [45] and its family members p63 and p73 [46]. iASPP was also identified as a binding partner and inhibitor of NF-κB subunit p65/RelA in cardiomyocytes and endothelium [47, 48]. Mice deficient for iASPP develop dilated cardiomyopathy with features of arrhythmogenic right ventricular cardiomyopathy (ARVC) with fibrosis and extensive immune infiltration [49]. Cardiomyocyte-specific and keratinocyte-specific iASPP-deficient mice develop ARVC or impaired cell adhesion and wound healing, respectively [50]. Although tissue-specific iASPP deletion largely phenocopies germline iASPP deletion, keratinocyte-specific iASPP-deficient mice have reduced cutaneous phenotype penetrance and severity, suggesting a possible non-cell autonomous role of iASPP. Disruption of immune homeostasis contributes to delayed wound healing [51–54] and increased inflammation is associated with more severe ARVC [55], suggesting that lymphocytes might provide this non-cell autonomous effect.

Given the recent implication of p53 in immune homeostasis, we hypothesized that iASPP regulates p53-mediated induction of immune tolerance by fundamentally altering the transcriptional profiles of dying cells, communicating the ultimate nature of cell death, whether physiologic or unnatural, to the responding immune system. We recently used genome-wide RNA-seq and p53 ChIP-seq analyses to discover iASPP regulation of a subset of p53 target genes involved in apoptosis and senescence [56]. Crystal structure analysis of the iASPP/p53 complex revealed that, unlike all other known cellular binding partners of p53, iASPP alters p53 target selectivity by binding to its L1 loop, which forms contacts with nucleotides defined by the iASPP/p53 sequence-specific signature. Here we demonstrate that perturbation of this iASPP/p53 complex in vivo disrupts the equilibrium between immunogenic versus tolerogenic cell death, with profound cell-intrinsic and non-cell-autonomous effects on the development of autoimmune disease and cancer.

## Results

### Experimental autoimmune encephalomyelitis is less severe in iASPP-deficient mice

To investigate the role of iASPP in immune homeostasis, we first characterized the peripheral immune system in *iASPP*^*−/−*^ mice [46] in the absence of immune challenge. *iASPP*^*−/−*^ mice had an increased proportion of CD4^+^FoxP3^+^ T_reg_ cells in the colonic lamina propria, inguinal lymph node, and liver (Fig. S1A–C) and peripheral CD4^+^ T cell populations in *iASPP*^*−/−*^ mice expressed elevated levels of PD-1 (Fig. S1D), suggesting that iASPP deficiency promotes peripheral T cell suppression and PD-1 expression. No increase in T_reg_ frequency was observed in the thymus, suggesting that iASPP doest not affect the development of natural T_reg_ (nT_reg_) cells. Interestingly, CD4^+^ and γδ T cells in the livers of aged *iASPP*^*−/−*^ mice expressed elevated levels of PD-1H (Fig. S1E), an immunosuppressive transcriptional target of p53 [17]. Given the increased proportion of T_reg_ cells observed in the periphery of *iASPP*^*−/−*^ mice, we examined whether iASPP deficiency promotes T_reg_ cell differentiation. Indeed, p53 induces T_reg_ cell differentiation and *FoxP3* transcription by directly binding its promoter [57, 58]. Naive CD4^+^CD44^*−*^CD62L^+^ (T_naive_) cells were purified and polarized toward T_reg_ or effector CD4^+^ subpopulations in vitro using Th1, Th2, Th17, or T_reg_ permissive conditions. iASPP deficiency decreased T_naive_ polarization to all subpopulations in vitro (Fig. S1F), suggesting that iASPP might indiscriminately modulate CD4^+^ polarization and differentiation. These findings suggest that the increased frequency of peripheral T_reg_ cells observed in *iASPP*^*−/−*^ mice arise through peripheral mechanisms of immune tolerance rather than dysregulated CD4^+^ differentiation.

**Fig. 1:**
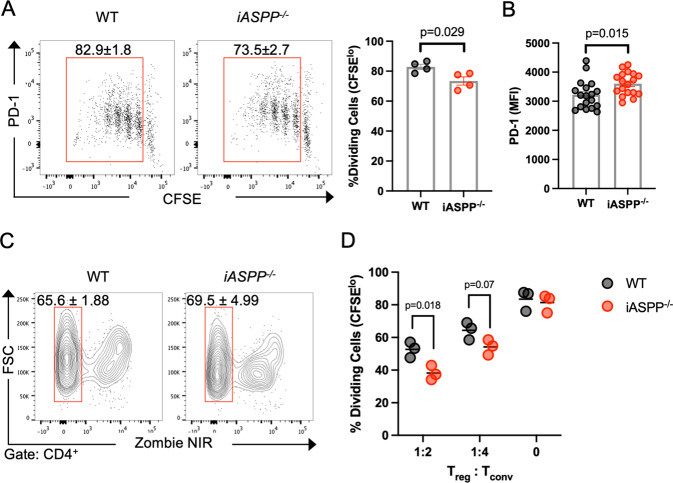
iASPP Deficiency Attenuates CD4+ T Cell Activation and Potentiates Treg Function. **A** Flow cytometry dot plots (left) and quantification (right) of CD4^+^ T cell division 72 h after anti-CD3 stimulation analyzed by CFSE dilution. **B** Flow cytometric quantification of PD-1 expression in CD4^+^ T cells in (**A**). MFI median fluorescent intensity. **C** Flow cytometry contour plots of live-dead stain of CD4^+^ T cells 72 h after anti-CD3 stimulation. **D** Flow cytometric quantification of suppressive activity of T_reg_ cells examined by cellular division of responder T_conv_ cells as measured by CFSE dilution 72 h after co-culture.

To determine functional consequences of iASPP-deficiency, we examined the proliferative and suppressive capacity of iASPP-deficient immune cells in vitro. *iASPP*^*−/−*^ CD4^+^ T cells had a decrease (*p* = 0.029) in proliferation measured by CFSE dilution in response to anti-CD3 (Fig. 1A) while expressing greater PD-1 (*p* = 0.015, Fig. 1B) without any decrease in viability (Fig. 1C), suggesting that iASPP deficiency limits CD4^+^ T cell proliferation in response to polyclonal TCR stimulation. Due to the apparent increased frequency of T_reg_ cells in *iASPP*^*−/−*^ mice, we examined the suppressive activity of *iASPP*^*−/−*^ T_reg_ cells in vitro by co-culturing CD4^+^CD25^*−*^ conventional T cells (T_conv_) and CD4^+^CD25^+^ T_reg_ cells purified from healthy WT or *iASPP*^*−/−*^ mice. *iASPP*^*−/−*^ T_reg_ cells exhibited greater suppressive capacity measured by decreased CFSE dilution (Fig. 1D), suggesting that iASPP expression in T_reg_ cells limits their suppressive potential. Together, these data suggest that cell-intrinsic iASPP expression may regulate CD4^+^ T cell proliferation and T_reg_ cell immunosuppression but not development. Increased T_reg_ cell frequency and CD4 T cell expression of PD-1H suggests that iASPP might regulate immune homeostasis through similar mechanisms as p53.

**Fig. 2:**
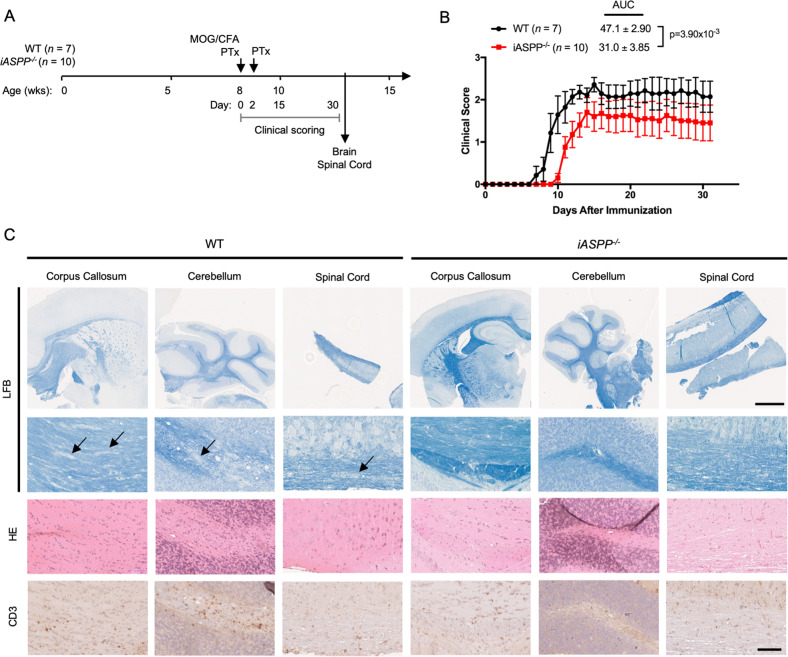
iASPP-Deficient Mice are Less Susceptible to Experimental Autoimmune Encephalitis. **A** Schematic of experimental autoimmune encephalomyelitis (EAE) model and experimental design. Mice were monitored until 30 days after immunization with MOG/CFA or until reaching primary endpoint of bilateral hind limb paralysis (clinical severity score = 3). MOG, 200 μg MOG_35-55_ peptide (MEVGWYRSPFSRVVHLYRNGK); CFA, Complete Freund’s Adjuvant supplemented with 400 μg H37Ra mycobacterium tuberculosis; PTx, 300 ng pertussis toxin. **B** Clinical course of MOG-induced EAE in WT (*n* = 7) or *iASPP*^*−/−*^ (*n* = 10) mice. AUC, area under the curve. **C** Luxol fast blue (LFB), HE, and CD3 immunohistochemistry stains of corpus callosum, cerebellum, and spinal cord isolated from WT (*n* = 4) or *iASPP*^*−/−*^ (*n* = 6) mice 30 days after immunization with MOG/CFA. Arrows indicate areas of demyelination. Scale, 1 mm (top) and 100 μm (bottom).

Given the influence of iASPP on tissue-resident T_reg_ counts, we hypothesized that iASPP^*−*/*−*^ mice would develop less severe autoimmune disease. To test this possibility, we utilized the myelin oligodendrocyte glycoprotein (MOG)-induced experimental autoimmune encephalomyelitis (EAE) model of T cell-mediated autoimmune demyelinating neuroinflammation (Fig. 2A). EAE was induced in WT (*n* = 7) or *iASPP*^*−/−*^ (*n* = 10) mice and clinical severity was measured until mice reached a clinical score of 3 (paralysis of both hind limbs), at which point mice were euthanized. Both WT and *iASPP*^*−/−*^ mice developed characteristic clinical features of EAE such as limp tail followed by limb paralysis and exhibited the expected relapsing-remitting disease course including initial attack and partial recovery after the peak phase of disease severity. However, *iASPP*^*−/−*^ mice had delayed disease onset (*p* = 0.015), decreased duration of disease (*p* = 2.2 × 10^–3^), and decreased overall severity of EAE (*p* = 3.9 × 10^–3^) (Figs. 2B, S2A, B). Histological examination of CNS pathology 30 days after EAE induction revealed that *iASPP*^*−/−*^ mice had less extensive demyelination measured by Luxol fast blue stain (Figs. 2C, S2C). *iASPP*^*−/−*^ mice also exhibited a decreased number of infiltrating T cells in the cerebellum (Figs. 2C, S2D). Therefore, iASPP deficiency appears to decrease CNS autoimmune disease severity.

Given the decrease in autoimmune neuroinflammation observed in *iASPP*^*−/−*^ mice, we analyzed potential sources of immunosuppression by performing flow cytometry on perfused whole brain hemispheres isolated from EAE mice 30 days after immunization. *iASPP*^*−/−*^ mice with EAE had >6-fold decrease (*p* = 0.03) in mean numbers of CNS-infiltrating CD4^+^ T cells (Fig. S2E). Functionality of the endogenous autoimmune T cell response in EAE was examined by measuring splenocyte proliferation in response to challenge with immunogen. Splenocytes isolated from EAE mice at peak disease severity (clinical score = 3) were stimulated with MOG_35-55_ peptide and extent of proliferation was measured using CFSE dilution. In concordance with the observed clinical disease course and flow cytometry immunophenotyping of CNS-infiltrating lymphocytes, splenocytes from *iASPP*^*−/−*^ mice with EAE were less responsive to in vitro immunogen challenge (Fig. S2F) and expressed increased amounts of PD-1H (Fig. S2G). Therefore, it appears that *iASPP*^*−/−*^ mice with EAE have a less potent autoimmune antigen-specific T cell response, suggesting that iASPP predisposes mice to more severe CNS autoimmune disease by fostering CD4^+^ T cell-mediated neuroinflammation.

### Pancreatic iASPP deficiency promotes an immunosuppressive tumor microenvironment

**Fig. 3:**
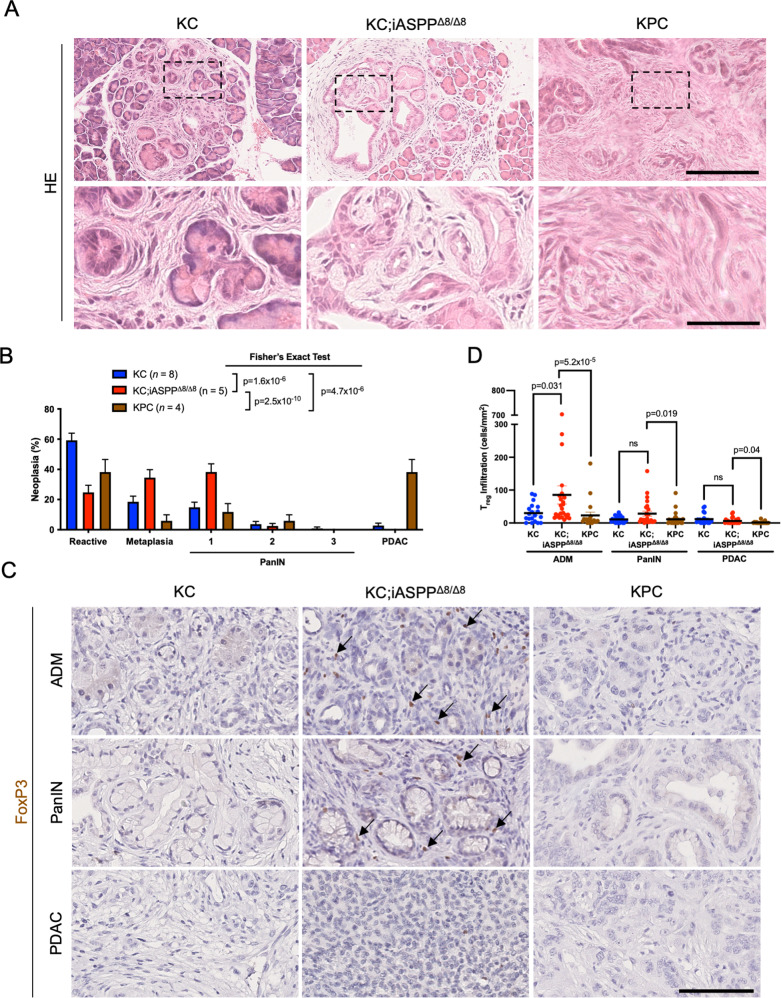
Pancreatic Tumors Lacking iASPP Have Increased Treg Infiltration. **A** Representative HE stains of pancreas isolated from 25-week-old *Kras*^*LSL−G12D/+*^*;Pdx1-Cre* (KC, *n* = 5), and *Kras*^*LSL−G12D/+*^*;iASPP*^*fl/fl*^*;Pdx1-Cre* (KC;iASPP^Δ8/Δ8^, *n* = 6), and *Kras*^*LSL−G12D/+*^*;Trp53*^*LSL−R172H/+*^*;Pdx1-Cre* (KPC, *n* = 5) mice. Higher magnification shown below. Scale, 200 μm (top) and 50 μm (bottom). **B** Proportion of reactive neoplasia, metaplasia, PanIN, versus PDAC of all pancreatic lesions in KC (*n* = 8, blue), KC;iASPP^Δ8/Δ8^ (*n* = 5, red), or KPC (*n* = 4, brown) mice. Data are sample proportion ± standard error of the sample proportion; Fisher’s exact test, *p* < 0.05 indicated on graph. **C** FoxP3 stains of pancreata isolated from KC (*n* = 5), KC;iASPP^Δ8/Δ8^ (*n* = 5), or KPC (*n* = 5) mice. Arrows indicate FoxP3+ cells. Scale, 100 μm. ADM acinar to ductal metaplasia, PanIN pancreatic intraepithelial neoplasia, PDAC pancreatic ductal adenocarcinoma. **D** Column scatter plot shows quantification of pancreatic neoplasia-infiltrating FoxP3^+^ T_reg_ cells per area.

Given the stimulatory effect of iASPP on CD4^+^ T cell autoimmunity and that iASPP can have varying functions in different tissues [50], we next examined whether epithelial cell-specific disruption of the iASPP/p53 complex could influence immunological tolerance in a non-cell autonomous manner by studying consequences of *iASPP* deletion in oncogenic Kras driven pancreatic neoplasias. As iASPP modulates p53 target selectivity [56] and p53 is tumor suppressive, we expected iASPP deletion to delay tumor development. However, tumor suppressor genes such as p53 and tumor immunosurveillance represent independent safeguards against cancer development whose relative importance remains unclear [59–62]. Although *Kras*^*LSL−G12D/+*^*;Trp53*^*LSL−R172H/+*^*;Pdx1-Cre* (KPC) mice developed the most accelerated progression to PDAC, *Kras*^*LSL−G12D/+*^*;iASPP*^*fl/fl*^*;Pdx1-Cre* (KC;iASPP^Δ8/Δ8^) mice paradoxically had advanced pancreatic neoplasia development compared to *Kras*^*LSL−G12D/+*^*;Pdx1-Cre* (KC) mice (Fig. 3A). While most KC neoplasias exhibited non-invasive epithelial proliferation and minimal cellular abnormalities after 25 weeks, most KC;iASPP^Δ8/Δ8^ pancreata harbored sites of acinar to ductal metaplasia (ADM) and pancreatic intraepithelial neoplasia (PanIN) (Fig. 3B), indicative of more advanced disease. KC;iASPP^Δ8/Δ8^ pancreatic neoplasias had increased infiltration of T_reg_ cells (Fig. 3C) and CD8 cells, but not CD4 cells (Fig. S3), suggesting that as in autoimmune disease, iASPP deficiency promotes T_reg_ cell infiltration over conventional CD4 T cell infiltration in pancreatic neoplasia. Importantly, KC;iASPP^Δ8/Δ8^ mice had greater extents of intratumoral T_reg_ cell infiltration than KC mice in early ADM lesions (Fig. 3D), demonstrating that increased presence of T_reg_ cells is dependent on loss of iASPP and not simply on more advanced pancreatic cancer. While increased T_reg_ cell infiltration was observed in KC;iASPP^Δ8/Δ8^ neoplasias and pre-cancerous lesions, sparse lymphocytic infiltration was observed in PDAC (Figs. 3D and S3), likely reflecting exclusion of lymphocytes from advanced pancreatic cancer [63]. Pancreatic CD4^+^ and CD8^+^ T cells exhibited a striking degree of exhaustion in KC;iASPP^Δ8/Δ8^ mice, including increased expression of PD-1 and Tim-3 (Fig. S3C), indicative of an exhausted T cell response in KC;iASPP^Δ8/Δ8^ mice. Together, these data suggest that advanced pancreatic neoplasia development observed in KC;iASPP^Δ8/Δ8^ mice is likely due to the emergence of an immunosuppressive tumor microenvironment that suppresses CD8^+^ anti-tumor cytotoxicity.

**Fig. 4:**
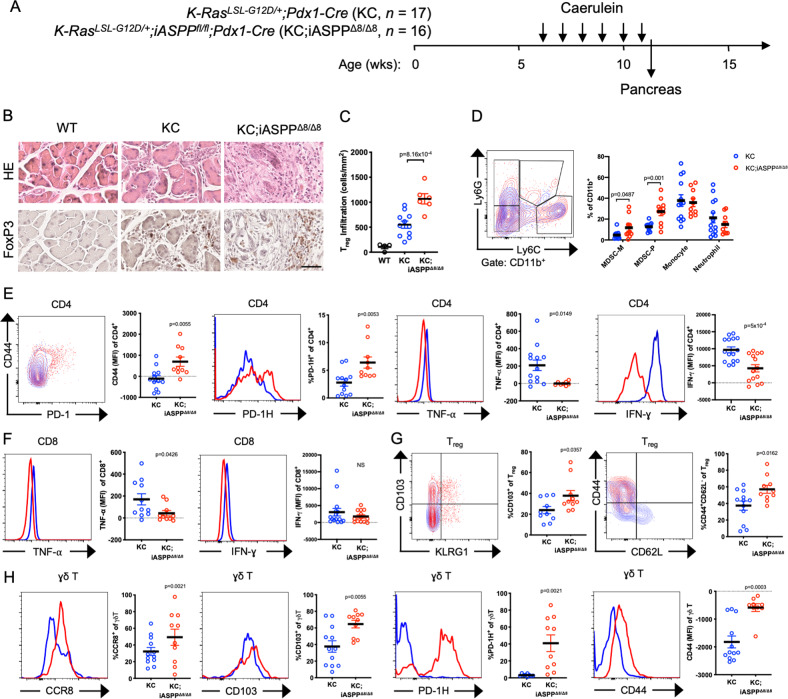
Regulatory and γδ T cells in iASPP Deficient Pancreatic Tumors Have an Activated Phenotype. **A** Schematic of chronic pancreatitis model and experimental design. Six-week-old WT (*n* = 5), *Kras*^*LSL−G12D/+*^*;Pdx1-Cre* (KC, *n* = 17), or *Kras*^*LSL−G12D/+*^*;iASPP*^*fl/fl*^*;Pdx1-Cre* (KC;iASPP^Δ8/Δ8^ *n* = 16) mice received six weekly doses of caerulein. Mice were sacrificed 48 h after the final dose and pancreata were collected for analysis. **B** HE, CD3, and FoxP3 immunohistochemistry stains of pancreata following six weekly doses of caerulein. Scale, 250 μm. **C** Column scatter plot shows quantification of FoxP3^+^ T_reg_ cells per neoplasia area. **D** Flow cytometry contour plot (left) and quantification (right) of gating strategy to identify MDSC-M, MDSC-P, monocytes, and neutrophils from KC (blue) or KC;iASPP^Δ8/Δ8^ (red) pancreata. **E** Flow cytometry plots (left) and quantification (right) of CD44, PD-1H, TNF-α, and IFN-γ expression in pancreata-infiltrating CD4^+^ cells from KC (blue) or KC;iASPP^Δ8/Δ8^ (red) pancreata. MFI median fluorescent intensity. **F** Flow cytometry histograms (left) and quantification (right) of TNF-α and IFN-γ expression in pancreata-infiltrating CD8^+^ cells from KC (blue) or KC;iASPP^Δ8/Δ8^ (red) pancreata. MFI median fluorescent intensity. **G** Flow cytometry contour plots (left) and quantification (right) of CD103, KLRG1, CD44 and CD62L expression in pancreata-infiltrating T_reg_ cells from KC (blue) or KC;iASPP^Δ8/Δ8^ (red) pancreata. MFI median fluorescent intensity. **H** Flow cytometry histograms of CCR8, CD103, PD-1H, and CD44 expression in pancreata-infiltrating γδ T cells from KC (blue) or KC;iASPP^Δ8/Δ8^ (red) pancreata.

To test whether iASPP deficient oncogenic Kras-driven pancreatic cancer maintains an immunosuppressive microenvironment in the presence of a canonical inflammatory insult [64], we subjected KC;iASPP^Δ8/Δ8^ mice to caerulein-induced chronic pancreatitis (Fig. 4A). Chronic pancreatitis resulted in substantial fibrosis and increased infiltration of T cells into the pancreas, and deletion of *iASPP* exhibited an even greater degree of reactive stroma (Fig. 4B) and T_reg_ infiltration (Fig. 4C) following caerulein insult. KC;iASPP^Δ8/Δ8^ pancreas-infiltrating DCs and macrophages expressed substantially increased amounts of MHCII and PD-1 (Fig. S4), and proportions of monocytic myeloid-derived suppressor cell (MDSC-M) and polymorphonuclear MDSC (MDSC-P) subpopulations were increased ~2-fold in KC;iASPP^Δ8/Δ8^ mice (Fig. 4D), indicative of the emergence of a potentially immunosuppressive population of APCs with *iASPP* deletion. Although KC;iASPP^Δ8/Δ8^ pancreas-infiltrating CD4^+^ T cells expressed increased CD44 (*p* = 5.5 × 10^–3^), they also had 2.3-fold increased expression of PD-1H (*p* = 5.3 × 10^–3^, Fig. 4E), suggesting that they might be subject to immunosuppression. Indeed, KC;iASPP^Δ8/Δ8^ pancreas-infiltrating CD4^+^ and CD8^+^ T cells secreted substantially less (*p* = 0.037) TNF-α and IFN-γ following chronic pancreatitis (Fig. 4E, F).

KC;iASPP^Δ8/Δ8^ pancreas-infiltrating T_reg_ cells and γδ T cells (*p* = 0.043) expressed higher amounts of CD44, CD103, and PD-1 (Fig. 4G, H). CD103 is a hallmark of activated tumor-infiltrating T_reg_ cells [65], but has not been previously described in γδ T cells. KC;iASPP^Δ8/Δ8^ pancreas-infiltrating γδ T cells expressed higher amounts of CCR8 (*p* = 2.1 × 10^–3^, Fig. 4H), whose expression strongly correlates with worse cancer prognosis [66, 67], and 41% of KC;iASPP^Δ8/Δ8^ pancreas-infiltrating γδ T cells expressed PD-1H (Fig. 4H). Flow cytometry performed on entire pancreata likely profiles both tumor-infiltrating lymphocytes and other components of the tumor microenvironment such as pancreatic lymph nodes and TLS. Indeed, tumor draining lymph nodes serve as unique reservoirs for anti-tumor immune responses [68, 69] and in pancreatic cancer contribute to an immunosuppressive tumor microenvironment [70, 71]. Deletion of *iASPP* in oncogenic Kras-driven pancreatic neoplasia elicited an immunosuppressive microenvironment that exhibited both canonical mechanisms of immunosuppression as well as previously undescribed T_reg_ and γδ T cell phenotypes.

### Oncogenic Kras driven lung adenocarcinoma is accelerated in iASPP-deficient lung tumors

**Fig. 5:**
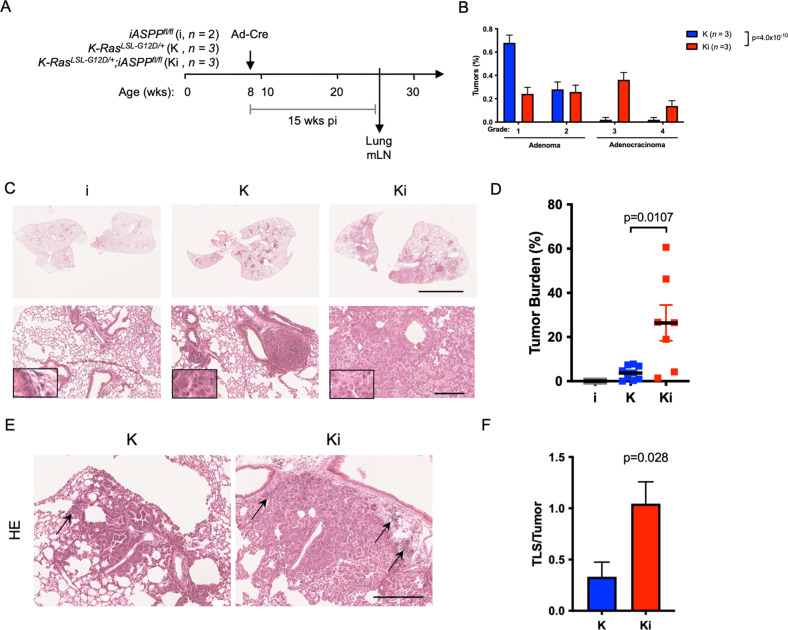
Oncogenic Kras Driven Lung Adenocarcinoma is Accelerated in Tumors Lacking iASPP. **A** Schematic of initiation of lung adenocarcinoma in *iASPP*^*fl/fl*^ (i, *n* = 2), *Kras*^*LSL−G12D/+*^ (K, *n* = 3), or *Kras*^*LSL−G12D/+*^*;iASPP*^*fl/fl*^ (Ki, *n* = 3) mice with intranasal inhalation of adenoviral Cre (Ad-Cre). Lungs and mediastinal lymph node (mLN) were collected 15 weeks post-infection (pi). **B** Proportion of lung tumors classified by histological grade. *P* value computed by Exact Contingency test. **C** HE stains of lung lobes 15 weeks after tumor initiation. Scale, 5 mm (top) and 250 μm (bottom). **D** Quantification of tumor burden of lung tumors in (**C**). Tumor burden was calculated as total tumor area divided by total area of each lung lobe. **E** HE stains of lung lobes 15 weeks after tumor initiation. Arrows indicate tertiary lymphoid structures (TLS). Scale, 250 μm. **F** Quantification of numbers of tertiary lymphoid structures (TLS) present per tumor from mice 15 weeks after tumor initiation.

Different tumor types employ vastly different mechanisms of immune escape and display varying responsiveness to immunotherapy [72, 73]. To determine whether iASPP-deficiency accelerates cancer development by attenuating anti-tumor immunity in a different cancer type, we employed the *Kras*^*LSL−G12D/+*^ model of lung adenocarcinoma (Fig. 5A). Intranasal delivery of adenovirus expressing Cre recombinase results in expression of oncogenic Kras^G12D^ in the lung epithelium, as oncogenic Kras^G12D^ can both promote tumorigenesis and the non-cell autonomous development of an inflammatory stroma via Myc, AP-1, and NF-κB [74–76]. In concordance with pancreatic neoplasia findings, 15 weeks after intranasal delivery of adenovirus expressing Cre recombinase, deletion of iASPP in *Kras*^*LSL−G12D/+*^*;iASPP*^*fl/fl*^ (Ki) mice elicited >7-fold increased tumor burden (*p* = 0.0107, Fig. 5B–D) and more advanced histological grade (*p* = 4.0 × 10^–10^) of lung cancer compared to *Kras*^*LSL−G12D/+*^ (K) mice (Fig. 5B). The majority (96%) of oncogenic Kras driven tumors were graded as adenomas, whereas 50% of Ki tumors had progressed to lung adenocarcinoma 15 weeks after initiation (Fig. 5B). These histological observations indicate that loss of iASPP accelerates oncogenic Kras driven lung adenocarcinoma, despite being a canonical inhibitor of p53 [45].

Tumor-associated lymphocytes were observed in oncogenic Kras-driven lung adenocarcinoma (Fig. 5E). However, most tumor-associated lymphocytes were retained in tertiary lymphoid structures (TLS) adjacent to lung tumors in Ki mice, while K mice had fewer TLSs present per tumor (Fig. 5F). TLSs are observed in human lung adenocarcinoma and correlate with positive prognosis [77–79], but have only been observed in mouse models of lung cancer that employ tumoral expression of strongly immunogenic antigens [80]. Therefore, Ki mice represent a distinct model of immunogenic lung adenocarcinoma, in which TLS surprisingly develop in mice with accelerated lung adenocarcinoma. Ki lung tumor-associated TLS contained elevated amounts of FoxP3^+^ T_regs_ (Fig. S5A), which might indirectly facilitate T_reg_ activation, suggesting that TLS formed in Ki mice might serve as sites of immunosuppression [81]. Flow cytometry of Ki tumor-bearing lungs revealed that lung tumor-associated CD4^+^, CD8^+^, and T_reg_ cells expressed increased PD-1, as did CD4^+^, CD8^+^, T_reg_, and myeloid cells in the tumor-draining mediastinal lymph node (Fig. S5B). Increased T_reg_ infiltration and expression of T cell markers of exhaustion are indicative of an immunosuppressive tumor microenvironment. As Ki mice possess a wild type immune system, the iASPP/p53 complex likely promotes cancer immunosurveillance through juxtacrine or paracrine signals from transformed cancer cells to the tumor microenvironment. Together these data suggest that tumoral iASPP-deficiency dampens the development of anti-tumor T cell responses while facilitating T_reg_ infiltration and activation, abating anti-tumor immune responses and accelerating lung cancer development.

### iASPP is a regulator of cell death adjuvanticity

**Fig. 6:**
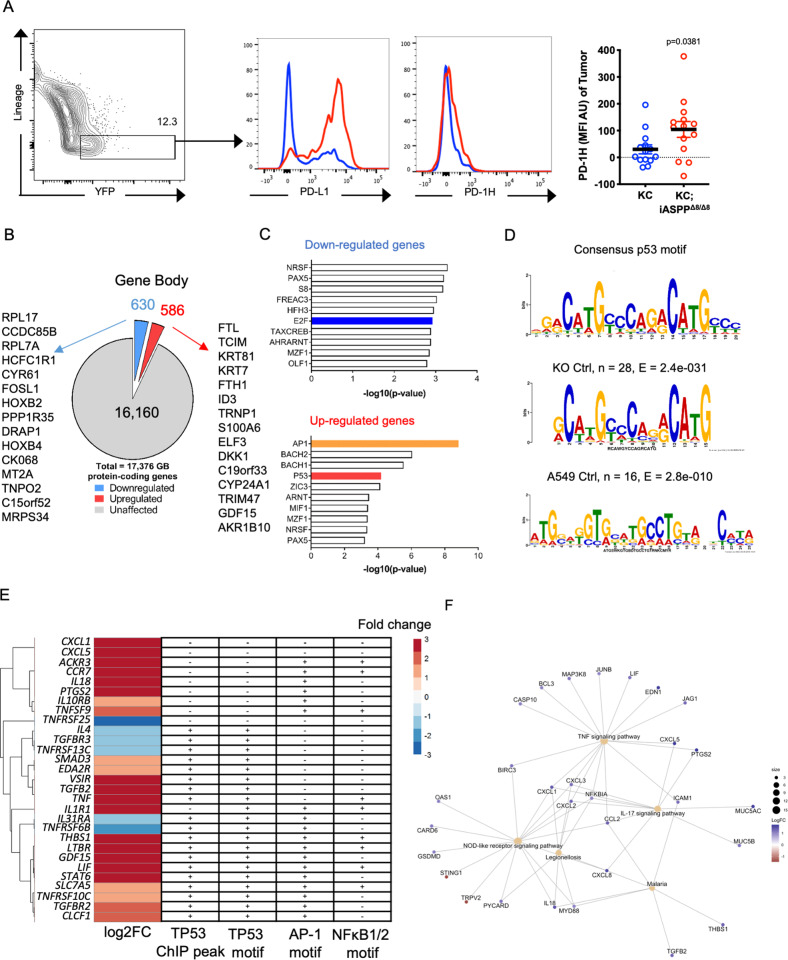
iASPP Deficiency Increases Cell-Autonomous Expression of Tolerogenic Mediators and DAMPs. **A** Flow cytometry histograms (left) and quantification (right) of PD-L1 and PD-1H expression by transformed KC (blue) or KC;iASPP^Δ8/Δ8^ (red) pancreatic tumor cells. MFI, median fluorescent intensity. **B** Differentially regulated genes in A549 cells following iASPP (*PPP1R13L*) knockdown. A gene is considered as differentially regulated when the mNET-seq and two ChIP-seq pol II signals in the gene body (TSS + 500 bp to poly(A) site) were >2-fold increased (upregulated, blue) or decreased (downregulated, red) in the knockdown cell line versus wild type cell line. The number of differentially expressed genes is indicated for each category. The genes indicated on the left and right sides are the 15 most downregulated or upregulated genes. **C** Ranked analysis of transcription factor binding sites in down (blue) or up (red)-regulated genes from the total pol II mNET-seq analysis in A549 cells following iASPP (*PPP1R13L*) knockdown. Transcription factor enrichment in up or down regulated genes was calculated using DAVID. **D** Motif analysis of indicated transcription factor binding sites in A549 cells following iASPP (*PPP1R13L*) knockdown. **E** Heatmap of log2FC in gene expression of indicated immune-related genes following iASPP (*PPP1R13L*) knockdown in A549 cells quantified by total pol II mNET-seq. The presence of a TP53 ChIP peak or TP53, AP-1, or NFκB 1/2 binding motif is indicated to the right for each gene. AP-1 and NFκB -1 motif presence was determined by GSEA and p53 motif presence was determined by MEME. **F** Network plot of gene ontology analysis of differentially expressed genes following iASPP (*PPP1R13L*) knockdown in A549 cells measured by total pol II mNET-seq. Tan circles represent gene sets and colored dots represent genes colored by log2FC.

Given that loss of *iASPP* promoted an immunosuppressive pancreatic tumor microenvironment that accelerated tumor development in a non-cell autonomous manner, we next asked what tumor cell-specific molecular mechanisms might be responsible for this immunosuppression. RNA-sequencing (RNA-seq) of pancreatic tumor lysates and principal component analysis revealed that KC;iASPP^Δ8/Δ8^ pancreata bear closer resemblance to KC than KPC pancreata (Fig. S6A) and express greater *Cd3, Cd4, Itgae, Pdcd1, Il10, Ccl5, Osm*, and *Cd274* (Fig. S6B), reflective of an immunosuppressive stromal reaction. Notably, this KC;iASPP^Δ8/Δ8^ -unique gene signature resembles the immunogenic subtype of human pancreatic cancer [82] for which there is currently no representative mouse model. KC;iASPP^Δ8/Δ8^ mice therefore represent a novel model of immunogenic pancreatic cancer. However, pancreatic tumor lysates include both transformed cancer cells and surrounding stroma. To identify the tumor cell-intrinsic factors responsible for promoting immunosuppression, we performed flow cytometry analysis of dissociated iASPP-deficient pancreata from KC or KC;iASPP^Δ8/Δ8^ mice with a *R26*^*LSL−EYFP/EYFP*^ allele. Transformed KC;iASPP^Δ8/Δ8^ pancreatic cells expressed higher amounts of PD-1H and PD-L1 in vivo (Fig. 6A), suggesting that these co-inhibitory receptors might be some of the tumor-intrinsic factors responsible for mitigating anti-tumor immunity following iASPP loss. Together these data suggest that transformed KC;iASPP^Δ8/Δ8^ tumor cells might modulate the surrounding tumor immune microenvironment via PD-1H juxtacrine signaling [17], activating PD-1H expression by local immunosuppressive T_reg_ and γδ T cells and suppressing functional anti-tumor adaptive immune responses.

Furthermore, knockout of the short isoform of *PPP1R13L* (gene name for iASPP) in A549 lung adenocarcinoma cells resulted in knockdown of full length iASPP as well as transcriptional changes consistent with immunomodulatory activity. First, we conducted total RNA polymerase II (pol II) ChIP-seq and mNET-seq to profile polymerase occupancy as a measure of active transcription for each gene following iASPP knockdown. iASPP knockdown resulted in increased polymerase occupancy at a *Vsir* (gene name for PD-1H) enhancer site with active chromatin marks (Fig. S6C), reaffirming that PD-1H might be an additional iASPP/p53-regulated mediator of immunological tolerance. Transcription factor motif analysis of pol II mNET-seq and ChIP-seq revealed that families of genes whose expression changed following *iASPP* knockdown contained TP53, AP-1, and/or NFκB1/2 motifs (Fig. 6B–D), suggesting that iASPP has transcription factor-specific modulatory ability. P53 binding was confirmed by P53 ChIP-seq (Fig. 6E). Gene set enrichment analysis confirmed that iASPP knockdown impacts P53, NFκB, and apoptosis-related genes (Figs. 6E, S6D), while gene network analysis provided insight into potential interactions between differentially-expressed genes (Fig. 6F). TNF and IL-17 signaling pathway activation is likely due to upstream AP-1 and NFκB signaling (Fig. 6E). These data suggest that iASPP-deficiency enacts broad genomic transcriptional changes by regulating p53 or other transcription factor target selectivity, increasing expression of both pro-inflammatory and immunosuppressive molecules normally expressed during physiologic cell death. Inflammatory molecule expression may facilitate tumorigenesis [83], while immunosuppressive molecules may antagonize cell corpse adjuvanticity and promote immunological tolerance.

## Discussion

Here we identify iASPP as a novel regulator of immunological tolerance. iASPP can promote autoimmune responses in vivo by limiting T_reg_ cell activation, while also enhancing cancer immunosurveillance by inhibiting T cell exhaustion and antagonizing T_reg_ and γδ T cell-mediated immune tolerance. These immune mechanisms are maintained by distinct cell types in which iASPP has complementary functions, namely oncogene transformed cancer cells and CD4^+^ T cells. In this model, iASPP and p53 function as regulators of transcriptional profiles intrinsic to dying cells that influence their adjuvanticity, thereby communicating to the immune system whether physiologic or unnatural cell death has occurred (Fig. S7).

iASPP paradoxically hindered tumor growth and development in vivo, contradicting the widely-accepted presumption that iASPP is a constitutive oncogene [84]. Although iASPP might function as an oncogene once tumors have escaped immunosurveillance, these data suggest that iASPP expression in developing tumors is critical for the establishment of a functional anti-tumor immune response. iASPP-deficient tumors displayed several novel mechanisms of immune escape not previously associated with lung or pancreatic cancer. MDSC-Ms and DCs expressed increased PD-1, whose expression on tumor-associated macrophages limits anti-tumor immunity in syngeneic tumor models [85]. γδ T cells contribute to PDAC development via an immunosuppressive phenotype [86], and were observed in both greater numbers and activation in iASPP-deficient tumors. Although iASPP deletion promoted a tolerogenic tumor microenvironment in lung and pancreatic tumors, the cell-intrinsic oncogenic properties of iASPP might dominate in other cancer types with less significant immunosurveillance. Not only does iASPP exhibit differential localization and function depending on cell type [46, 49, 87, 88], but neoplastic cells also employ diverse mechanisms of immune escape depending on tissue context [89]. Although we focus on the effects of iASPP deletion on lymphocytes here, iASPP might exert non-cell autonomous functions on other cell types as well, such as cancer-associated fibroblasts in pancreatic neoplasias, or astrocytes and microglia in EAE. Further experiments in different mouse models of cancer are necessary to assess the universality of iASPP non-cell autonomous tumor-suppression.

Mechanistically, as iASPP was selectively deleted in neoplastic cells in mouse models of lung adenocarcinoma and pancreatic cancer, transformed cells themselves must modulate the activation of an otherwise wild-type immune system, likely through juxtacrine or paracrine signaling. We suggest that iASPP functions by inhibiting p53-mediated immunosuppression, particularly by modulating p53 transcriptional promotion of tolerance following physiologic cell death. Fas is one of the most significantly iASPP-regulated p53 target genes [56], and its expression on apoptotic cells promotes tolerogenic phagocytosis [35, 90]. Loss of function or dominant-negative mutations in Fas/FasL are associated with autoimmune disease [28, 91–98] and Fas overexpression facilitated tumorigenesis in vivo in a non-cell autonomous manner by promoting MDSC and T_reg_ cell recruitment [99]. Interestingly, T_reg_ cells express decreased FasL and are resistant to Fas-mediated apoptosis [100], potentially explaining their relative abundance we observed in vivo. PD-1H was another p53 target overexpressed in iASPP-deficient tumor cells as well as responding lymphocytes, suggesting that iASPP might inhibit p53 transcriptional activation of PD-1H and its subsequent juxtacrine signaling [17]. *iASPP*^*−/−*^ mice exhibited significantly increased immune tolerance that largely phenocopied the consequences of treatment with a PD-1H agonist [101]. In addition to upregulation via p53 in the setting of programmed cell death [17, 102], Fas and PD-1H both promote immunological tolerance in a non-cell autonomous manner by acting as negative regulators of cell death-related adjuvanticity to responding phagocytes [16, 34, 90]. Without iASPP-mediated modulation of target selectivity, p53 might also promote tumorigenesis via tolerogenic or inflammatory DAMPs, such as PGE2 and IL-1α [103, 104], and general immunosuppression via senescence or tolerogenic molecules such as CD47, MFG-E8, or thrombospondin [105, 106]. Indeed, iASPP-deficient cells increased expression of inflammatory cytokines *S100a8* and *S100a9*, which are also expressed by apoptotic cancer cells and can promote metastasis [107, 108]. Further investigation is necessary to determine if these potential cell-intrinsic mechanisms, in addition to Fas and PD-1H, are responsible for promoting the non-cell autonomous tolerance observed in vivo.

Although the data presented here suggest that iASPP promotes adjuvanticity by antagonizing p53-mediated tolerogenic phagocytosis, both iASPP and p53 have additional independent effects on the immune system. p53 functions in a positive feedback loop to drive type I IFN-mediated antiviral immunity [109], and can suppress STAT1 and NF-κB mediated inflammation [32]. p53-dependent activation of Batf3 in intratumoral monocytic MDSCs is essential for the development of anti-tumoral Ly6c^+^CD103^+^ DCs [110]. iASPP can inhibit NF-κB subunit RelA/p65-mediated inflammation and pro-proliferative functions [47, 111], even in the absence of p53 [48, 112], indicating that iASPP inhibition of RelA/p65 is functionally orthogonal to its inhibition of p53. RelA/p65 expression is essential for maintenance of T_reg_ function, identity, and immune tolerance [113]. Therefore, iASPP inhibition of NF-κB mediated immune tolerance in T_reg_ cells is a potential p53-independent mechanism of iASPP immunological influence, although iASPP likely also has T_reg_ cell-extrinsic roles as tumor-specific deletion of iASPP promoted tolerogenic immune responses. iASPP regulates p63 target selectivity in keratinocytes with differential binding to promoters with AP-1 versus E2F motifs [114], suggesting that cross-talk between iASPP and other co-factors might be responsible for cell type-specific transcriptional regulation. Indeed, we identified AP-1, P53, E2F, and NF-κB binding motifs amongst inflammatory genes differentially expressed in iASPP deficient A549 lung cancer cells. Ultimately, the multifaceted roles of iASPP likely come together to influence the adaptive immune system, as chronic inflammation can both promote tumorigenesis and induce immunosuppression [83, 115–117].

This study identifies iASPP as a novel component of the maintenance of immune homeostasis between tolerance and autoimmunity. Loss of iASPP in cancer cells, CD4^+^ T cells, or with germline deletion elicited strikingly elevated immune tolerance. These data suggest that iASPP is a previously unidentified regulator of the immunological phenotypes of dying cells and thereby orchestrates the ensuing innate and adaptive immune responses to cell death. Although many signaling mediators such as DAMPs and ‘eat-me signals’ have been identified, the underlying pathways responsible for discriminating between physiologic apoptosis and unnatural cell death have remained previously uncharacterized. While this study demonstrates iASPP promotion of non-physiologic cell death adjuvanticity in the contexts of autoimmune disease and cancer, the normal physiologic role of iASPP in providing adjuvant co-stimulation remains unclear. Investigation of iASPP and its regulatory mediators provides a previously unappreciated opportunity to identify immunological therapeutic targets with implications for multiple diseases.

## Materials and methods

### Mice

*iASPP*^*−/−*^ [46, 49]*, iASPP*^*fl/fl*^ [46]*, ASPP2*^*fl/fl*^ [118], *Kras*^*LSL−G12D*^ [119], *Pdx1-Cre* [120], *Trp53*^*LSL−R172H/+*^ [121], *Rosa26*^*LSL−EYFP*^ [122], and *Rosa26*^*CreER*^ [123] mice have been previously described. *iASPP*^*fl/fl*^ mice were back-crossed into a C57BL/6 background strain prior to being bred with *Pdx1-Cre* and *Rosa26*^*LSL−EYFP/EYFP*^ mice (Jackson Laboratory) to generate *iASPP*^*fl/fl*^*;Rosa26*^*LSL−EYFP/EYFP*^ (i) and *iASPP*^*fl/fl*^*;Pdx1-Cre;Rosa26*^*LSL−EYFP/EYFP*^ (iC) mice. *Kras*^*LSL−G12D/+*^*;Rosa26*^*LSL−EYFP/EYFP*^ (K)*, Kras*^*LSL−G12D/+*^*;Pdx1-Cre;Rosa26*^*LSL−EYFP/EYFP*^ (KC), *Kras*^*LSL−G12D/+*^*;iASPP*^*fl/fl*^*;Rosa26*^*LSL−EYFP/EYFP*^ (Ki), and *Kras*^*LSL−G12D/+*^*; iASPP*^*fl/fl*^*;Pdx1-Cre;Rosa26*^*LSL−EYFP/EYFP*^ (KC;iASPP^Δ8/Δ8^) mice were generated by crossing *Kras*^*LSL−G12D*^ heterozygous mice with iC or *Pdx1-Cre* mice. *Kras*^*LSL−G12D/+*^*;Trp53*^*LSL−R172H/+*^ (KP) and *Kras*^*LSL−G12D/+*^*;Trp53*^*LSL−R172H/+*^*;Pdx1-Cre;Rosa26*^*LSL−EYFP/EYFP*^ (KPC) mice were generated by crossing KC mice with *Trp53*^*LSL−R172H*^ heterozygous mice. Mice heterozygous for deletion of exon 8 of *Ppp1R13l* (*iASPP*^*Δ8/+*^) were used to generate *iASPP*^*−/−*^ mice and littermate wild type controls. The health of mice was monitored daily by veterinary staff at the Wellcome Trust Centre for Human Genetics (WTCHG) at the University of Oxford. No sample size calculation estimate or randomization were performed. Both male and female mice were used, mice were gender and age-matched within experiments where possible, and adult mice (>8 weeks old) were used for all experiments. Experimental and control mice were co-housed whenever appropriate. All mice were housed and all procedures were performed at the WTCHG at the University of Oxford. All animal studies described in this study were performed in accordance with guidelines provided by the University of Oxford Institutional Animal Care and Use Committee. Procedures were performed under the Home Office Animal Scientific Procedures Act 1986 guidelines (license PPL 30/3451 and PPL 30/3096).

### PCR genotyping

After mice were weaned, DNA was isolated from ear clips by incubation in TDB (50 mM KCl, 10 mM Tris-HCl, 0.1% Triton-X100 in H_2_O) with 0.4 mg/mL Proteinase K (Qiagen) for 2 h at 55 °C, then 10 min at 95 °C. *Pdx1-Cre* and *Rosa26*^*CreER*^ mice were genotyped using the following primers: Cre-F, 5′-CATTTGGGCCAGCTAAACAT-3′; Cre-B, 5′-ATTCTCCCACCGTCAGTACG-3′; Actin-F, 5′-GGTGTCATGGTAGGTATGGGT-3′; and Actin-B, 5′-CGCACAATCTCACGTTCAG-3′. i*ASPP2* ^*fl/fl*^ mice were genotyped using the following primers: FRANT9, 5′-GGGTAGGAAAAAGGGCTGAG-3′; FLP2, 5′-CCGAATTGGAGAAGTGAAGC-3′. *Kras*^*LSL−G12D/+*^ mice were genotyped using the following primers: Y116, 5′-TCCGAATTCAGTGACTACAGATG-3′; Y117, 5′-CTAGCCACCATGGCTTGAGT-3′; Y118, 5′-ATGTCTTTCCCCAGCACAGT-3′. *Rosa26*^*LSL−EYFP/EYFP*^ mice were genotyped using the following primers: YFPc, 5′-AAAGTCGCTCTGAGTTGTTAT-3′ and YFPwt, 5′-GGAGCGGGAGAAATGGATATG-3′; or YFPc and YFPmut, 5′-AAGACCGCGAAGAGTTTGTC-3′. *Trp53*^*LSL−R172H/+*^ mice were genotyped using the following primers: p53u, 5′-CTTGGAGACATAGCCACACTG-3′ and p53wt, 5′-TTACACATCCAGCCTCTGTGG-3′; or p53u and p53mut, 5′-AGCTAGCCACCATGGCTTGAGTAAGTCTGCA-3′. *iASPP*^*−/−*^ mice were genotyped using the following primers: I8-2, 5′- ACAGGCAGCTACTGGTATTC-3′ and E8-2, 5′-AGAGCAGCCTCAGAGCATGG-3′; or I8-2 and FLP2.

PCR reactions were performed on 0.5 μL of sample DNA using Green *Taq* DNA Polymerase (GenScript) according to manufacturer’s protocols. The *Pdx1-Cre* and *Rosa26*^*CreER*^ allele yields a 308 bp product while the wild type allele yields a 753 bp product from primers Cre-F, Cre-B, Actin-F, and Actin-B. The *ASPP2* ^*fl/fl*^ allele yields a 203 bp product while the wild type allele yields a 169 bp product from primers SDL1s and SDL2s. The *iASPP* ^*fl/fl*^ allele yields a 400 bp product while the wild type allele yields a 265 bp product from primers FRANT9 and FLP2. The *Kras*^*LSL−G12D*^ allele yields a 327 bp product while the wild type allele yields a 450 bp product from primers Y116, Y117, and Y118. The *Rosa26*^*LSL−EYFP*^ allele yields a 320 bp product from primers YFPc and YFPmut, while the wild type allele yields a 600 bp product from primers YFPc and YFPwt. The *Trp53*^*LSL−R172H*^ allele yields a 270 bp product from primers p53u and p53mut, while the wild type allele yields a 166 bp product from primers p53u and p53wt. The *iASPP*^*−/−*^ allele yields a 700 bp product from primers I8-2 and FLP2, while the wild type allele yields a 600 bp product from primers I8-2 and E8-2.

### Preparation of mouse T cell populations

Spleens from 6–8-week-old mice were dissociated by mechanical dissociation using the plunger of a 2.5 mL syringe on a 70 μm cell strainer (Falcon). Red blood cells were lysed with ACK Lysis buffer (150 mM NH_4_Cl, 10 mM KHCO_3_, 0.1 mM EDTA in H_2_O, pH 7.4) for 2 min at RT. Following centrifugation cells were resuspended in RPMI 1640 (Gibco) supplemented with 1% heat-inactivated fetal calf serum (HI-FCS), 2 mM L-glutamine, 100 U/mL penicillin, and 100 μg/mL streptomyocin and counted using a hemocytometer. Splenocytes were then washed and resuspended in 0.5% BSA and 2 mM EDTA in PBS at 1 × 10^7^ cells/mL for magnetic-activated cell sorting (MACS). CD4^+^ T cells were isolated by negative selection using a CD4^+^ T Cell Isolation Kit and LS columns (Miltenyi Bitoec), naive CD4^+^CD44^*−*^CD62L^+^ T cells were isolated using a Naive CD4^+^ T Cell Isolation Kit and LS columns (Miltenyi Biotec), and CD4^+^CD25^*−*^ conventional T cells (T_conv_) and CD4^+^CD25^+^ regulatory T cells (T_reg_) were isolated by sequential negative and positive selection using a CD4^+^CD25^+^ Regulatory T Cell Isolation Kit and LD and MS columns (Miltenyi Biotec) according to manufacturer’s protocols.

### In vitro T cell activation

CD4^+^ T cells were washed and resuspended at 1 × 10^7^ cells/mL in PBS and labeled with 5 μM CellTrace carboxyfluorescein succinimidyl ester (CFSE) Cell Proliferation Dye (Life Technologies) for 10 min at 37 °C. Staining was quenched with five volumes of HI-FCS and cells were resuspended at 5 × 10^5^ cells/mL in RPMI 1640 supplemented with 10% HI-FCS, 10 mM HEPES (Life Technologies), 50 μM β-mercaptoethanol (Sigma), 2 mM L-glutamine, 100 U/mL penicillin, and 100 μg/mL streptomyocin with or without 40 ng/mL murine IL-2 (PeproTech), Cells were stimulated with indicated concentrations of plate-bound anti-CD3ε mAb (145-2C11, BioLegend) at 200 μL/well in a 96-well plate, and CFSE dilution was measured by flow cytometry after 72 h.

### T_reg_ suppression assay

CD4^+^CD25^*−*^ T_conv_ cells were washed and resuspended at 1.5 × 10^6^ cells/mL in PBS and labeled with 5 μM CFSE for 10 min at 37 °C. Staining was quenched with five volumes of HI-FCS and cells were resuspended at 5 × 10^5^ cells/mL in RPMI 1640 supplemented with 10% HI-FCS, 10 mM HEPES (Life Technologies), 50 μM β-mercaptoethanol (Sigma), 1 mM sodium pyruvate (Life Technologies), 100 mM non-essential amino acids (Life Technologies), 2 mM L-glutamine, 100 U/mL penicillin, and 100 μg/mL streptomyocin. Control splenocytes were resuspended at 1 × 10^7^ cells/mL and exposed to 30 Gy gamma irradiation and labeled with soluble 1 μg/mL anti-CD3ε mAb. CD4^+^CD25^+^ T_reg_ cells were co-cultured at indicated ratios with 5 × 10^4^ irradiated splenocytes and 2.5 × 10^4^ T_conv_ cells/well in round-bottom 96-well plates (Corning). After 72 h cells were washed and CFSE dilution was measured by flow cytometry.

### CD4 differentiation assay

Naive CD4^+^CD44^*−*^CD62L^+^ T cells were resuspended at 1 × 10^6^ cells/mL in RPMI 1640 supplemented with 10% HI-FCS, 50 μM β-mercaptoethanol, 2 mM L-glutamine, 100 U/mL penicillin, and 100 μg/mL streptomyocin and stimulated with plate-bound 1 μg/mL anti-CD3ε mAb and 1 μg/mL anti-CD28 (37.51, BioLegend) at 200 μL/well in a 96-well plate. Cells were polarized as indicated using the following culture conditions: Th0, 5 μg/mL anti-IL-4 mAb (11B11, BioLegend) and 5 μg/mL anti-IFN-γ mAb (XMG1.2, BioLegend); Th1, 10 ng/mL murine IL-12 (PeproTech) and 5 μg/mL anti-IL-4 mAb; Th2, 10 ng/mL murine IL-4 (PeproTech) and 5 μg/mL anti-IFN-γ mAb; Th17, 5 μg/mL anti-IL-4 mAb, 5 μg/mL anti-IFN-γ mAb, 10 ng/mL human IL-23 (PeproTech), 1 ng/mL human TGF-β (PeproTech), and 5 ng/mL murine IL-6 (PeproTech); T_reg_, 1 ng/mL human TGF-β, 5 μg/mL anti-IL-4 mAb, and 10 μg/mL anti-IFN-γ mAb. After 72 hr cells were analyzed by intracellular flow cytometry for CD4^+^ Th subset polarization.

### Experimental autoimmune encephalomyelitis

Experimental autoimmune encephalomyelitis (EAE) was induced in 8–10-week-old female mice by bilateral subcutaneous (s.c.) injection of a total of 200 μg MOG_35-55_ (MEVGWYRSPFSRVVHLYRNGK, Severn Biotech) emulsified 1:1 in complete Freund’s adjuvant supplemented with 400 μg H37Ra mycobacterium tuberculosis (CFA, Chondrex) in incomplete Freund’s adjuvant (Sigma). Immunization was boosted immediately and 48 h after s.c. injection with 300 ng pertussis toxin (PTx, Merck Chemicals) by i.v. injection. Mice were weighed and neurological impairment was measured daily in a genotype-blind fashion using the following clinical severity scoring system: 0, healthy; 0.5, partial tail limpness; 1, tail paralysis; 1.5, righting reflex weakness; 2, hind limb weakness; 2.5, paralysis of one hind limb; 3, paralysis of both hind limbs; 3.5 hind limb paralysis and paralysis of one fore limb; 4, quadriplegia; 5, moribund. When mice reached a score of 1, cages were changed to dry bedding and wet mash was placed on the cage floor. Under recommendation of the animal ethics committee, mice were humanely euthanized when they reached a clinical score greater than or equal to 3. Any mice exhibiting adverse signs related to EAE such as >20% weight loss, labored breathing, or inability to access food were humanely euthanized. Mice remaining alive 30 days after immunization were sacrificed and spleens, brains, spinal cords, iLNs, and blood were collected for analysis.

### MOG_35-55_ stimulation assay

Splenocytes were washed and resuspended at 1 × 10^7^ cells/mL in PBS and labeled with 5 μM CFSE for 10 min at 37 °C. Staining was quenched with five volumes of HI-FCS and cells were resuspended at 1 × 10^6^ cells/mL in RPMI 1640 supplemented with 40 ng/mL murine recombinant IL-2, 10% HI-FCS, 2 mM L-glutamine, 100 U/mL penicillin, and 100 μg/mL streptomyocin. Splenocytes were then stimulated with indicated concentrations of MOG_35-55_ peptide for 72 h, and CFSE dilution was measured by flow cytometry.

### Adenoviral infection

Autochthonous lung adenocarcinoma was initiated in 9–13 week old mice by intranasal infection with 2.5 × 10^7^ PFU adenovirus as previously described [124, 125]. Briefly, Ad-CMV-iCre (Vector Biolabs) was resuspended in 50 μL/mouse Opti-MEM (Life Technologies) with 10 mM calcium chloride. Mice were anesthetized with isoflurane until respiration rate reached 2 respirations/sec and 25 μL of adenovirus suspension was administered dropwise to a single nostril. After 5 min of recovery, adenoviral administration was repeated on the same nostril. Mice were monitored daily and any mice exhibiting adverse signs related to tumor burden such as hunched posture, labored respiration, or >15% weight loss were humanely euthanized. All mice were sacrificed 15 weeks after tumor initiation and spleens, lungs, livers, and mediastinal lymph nodes (mLN) were collected for analysis.

### Caerulein-induced chronic pancreatitis

Chronic pancreatitis was induced in 6–8-week-old mice by six weekly intraperitoneal (i.p.) injections of 250 μg/kg of caerulein (Sigma) in PBS. Approximately equal numbers of male and female mice were used in all experimental groups. Mice were monitored daily and any mice exhibiting adverse signs related to chronic pancreatitis or tumor burden such as hunched posture, piloerection, >15% weight loss, palpable abdominal masses, lethargy, diarrhea, rectal bleeding, ulcerated tumors, tumors interfering with locomotion, feeding, breathing, or vision, genital prolapse, or dermatitis were humanely euthanized. Mice were sacrificed 48 h following the final caerulein injection and spleens, pancreas, lungs, and livers were collected for analysis.

### Tissue collection

Mice were sacrificed by cervical dislocation, transcardially perfused with 40 mL PBS and tissues were allocated for immunohistochemistry (IHC), immunofluorescence, flow cytometry, RNA isolation, in vitro assays, or tissue lysate preparation. In cases where tissues were only taken for IHC or immunofluorescence, mice were subsequently perfused with paraformaldehyde-lysine-periodate fixative (PLP; 50 mM phosphate buffer with 1% paraformaldehyde (Sigma), 0.1 M L-lysine buffer (pH 7.4), 0.2% NaIO_4_ (Sigma)). Tissue lysates were prepared by homogenizing organs in 50 μL/mg RPMI with 1% Triton X-100 (Sigma) with a rotor-stator tissue homogenizer for 15 s, followed by centrifugation at 5000 *g* for 10 min. RNA was prepared from snap-frozen tissues by grinding with mortar and pestle in liquid nitrogen, followed by tissue homogenization in 600 μL Buffer RLT (Qiagen) with β-mercaptoethanol and RNA isolation using the RNeasy Mini Kit according to manufacturer’s protocols.

### Immunohistochemistry

Isolated tissues were individually perfused with PBS followed by PLP. Tissues were fixed overnight in PLP at 4 °C and stored in 70% ethanol until processing. PLP-fixed tissues were dehydrated overnight using a Shandon Excelsior Tissue Processor (Thermo), embedded in paraffin, sectioned at a thickness of 4 μm, and stained with haematoxylin and eosin using standard methods. For IHC, slides were dewaxed and antigen retrieval was performed by boiling in Tris-EDTA (pH 9) and 0.5% Tween-20 (Sigma) for 3 min at 120 °C in a pressure cooker. Slides were then cooled, washed with dH_2_O, and endogenous peroxidases were blocked with 3% hydrogen peroxide (Sigma) in methanol (Sigma) for 15 min at RT. Tissues were blocked with 5% normal goat (Vector Laboratories) or donkey serum (Sigma) in PBS, and stained with primary antibody overnight at 4 °C followed by biotinylated secondary anti-rabbit, anti-hamster, anti-rat, or anti-goat (Vector Laboratories) for 30 min at RT and Vectastain Elite ABC (Vector Laboratories) for 30 min at RT. Staining was visualized using ImmPACT DAB Peroxidase (Vector Laboratories) using manufacturer’s protocols, and slides were counterstained with haematoxylin for 4 s, dehydrated to Histo-Clear II (National Diagnostics), and coverslipped using VectaMount Permanent Mounting Medium (Vector Laboratories). The following primary antibodies were used for IHC: rabbit anti-CD3 (ab5690; 1:200, 4 min develop), rat anti-FoxP3 (FJK-16s, eBioscience; 1:50, 30 min develop). Images were obtained using a NanoZoomer S210 (Hamamatsu) and analyzed using NDP.view2 software, or using a Leica DM5500B and analyzed using Fiji.

Luxol fast blue staining was performed by incubating sections in 0.1% luxol fast blue (Fisher) in 95% ethanol with 0.5% glacial acetic acid overnight at 56 °C. Slides were then differentiated in 0.05% lithium carbonate (Scientific Laboratory Supplies Ltd) in dH_2_O for 30 s, then 70% ethanol for 30 s, and counterstained with 0.1% cresyl violet acetate (MP Biomedicals) in dH_2_O with glacial acetic acid.

### Histological quantification

Grading of pancreatic neoplasias was performed in a blind fashion as described [126]. Briefly, pancreatic neoplasias were categorized as metaplasia (ADM) when exhibiting non-invasive epithelial proliferation and cellular abnormalities, PanIN when glandular epithelial proliferation was confined to pancreatic ducts, and pancreatic ductal adenocarcinoma (PDAC) when exhibiting ductal differentiation and penetration of the basement membrane. Lung tumor grading was performed in a blind fashion as previously described [125, 127, 128]. Briefly, lung adenomas were identified by prominent nucleoli, while lung adenocarcinomas were graded by extent of nuclear atypia, cellular pleomorphism, invasiveness, mitotic index, cellular heterogeneity, and desmoplasia.

IHC images were blinded using blindanalysis v1.0 [129]. T_reg_ infiltration was assessed in a blind fashion by counting the number of FoxP3^+^ nuclei present in IHC images of tumors. Tumor area was measured in Fiji by outlining the perimeter of tumors and calculating the enclosed area. For analysis, average T_reg_ infiltration was calculated by diving the total number of infiltrating FoxP3^+^ cells by the total tumor area across three or more fields of view per mouse. Tumor burden was measured per lung lobe in NDPview.2 by outlining the area of individual tumors and of individual lobes. CD3 infiltration into the CNS was assessed in a blind fashion by counting the number of CD3 cells present in in IHC images of brain and spinal cord. Average CD3 infiltration was calculated by averaging across three or more fields of view for each mouse. Demyelination was quantified by measuring Luxol fast blue intensity using the Mean Gray Value function in Fiji.

### Flow cytometry

Lymph nodes were dissociated by mechanical dissociation between two frosted microscope slides (Fisher). Spleens, thymus, livers, and brains were dissociated by mechanical dissociation using the plunger of a 2.5 mL syringe on a 70 μm cell strainer (Falcon). Livers and brains were further processed by centrifugation in a Percoll (VWR) gradient at 700 *g* for 25 min with no brake, and lymphocytes were isolated from the 40–70% Percoll interface.

Lungs, pancreas, kidneys, and hearts were placed in 5 mL collagenase buffer (125 U/mL collagenase IV (Worthington), 40 U/mL DNaseI (Roche), 25 mM HEPES, and 0.5% HI-FCS in 1X HBSS) in C tubes (Miltenyi Biotec). Tissues were dissociated using program “m_imp_tumor_01” for lungs, “m_imp_tumor_04” for pancreas, “multi_E_01” for kidneys, or “m_heart_01” for hearts on a gentleMACS dissociator (Miltenyi Biotec), incubated at 37 °C for 40 min with gentle agitation, then dissociation was repeated using program “m_imp_tumor_01” for lungs, “m_imp_tumor_04” for pancreas, “multi_E_02” for kidneys, or “m_heart_02” for hearts. Following dissociation cells were filtered through a 100 μm cell strainer (Falcon).

Colons were cut longitudinally and washed in PBS, then cut into 5 mm pieces and incubated in 5 mM EDTA in PBS for 15 min at 37 °C. Washed tissues were then placed in 5 mL of RPMI 1640 supplemented with 5% HI-FCS, 20 mM HEPES, 100 U/mL penicillin, 100 μg/mL streptomyocin, and 100 U/mL collagenase VIII (Sigma) in C tubes. Tissues were dissociated using program “m_imp_intestine_01”, incubated for 60 min at 37 °C with gentle agitation, dissociated again using program “m_imp_intestine_01”, and filtered through a 100 μm cell strainer. Cells were then resuspended and centrifuged in a Percoll gradient at 700 *g* for 25 min with no brake, and lymphocytes were isolated from the 40 to 70% Percoll interface.

Red blood cells were lysed with ACK Lysis buffer for 2 min at RT and resuspended in RPMI 1640 supplemented with 1% HI-FCS, 2 mM L-glutamine, 100 U/mL penicillin, and 100 μg/mL streptomyocin, counted, and adjusted to 1 × 10^7^ cells/mL. 3 × 10^3^ to 1 × 10^7^ cells were Fc receptor blocked with rat anti-CD16/CD32 (2.4G2, BD Biosciences; 1:100) for 5 min, and stained for 30 min on ice in round-bottom 96-well plates with Zombie Green Viability Dye (BioLegend; 1:1000) or Zombie NIR Viability Dye (BioLegend; 1:1000) and directly-conjugated antibodies in 0.5% HI-FCS and 0.05% sodium azide in PBS. Following staining cells were fixed with Fixation/Permeabilization Buffer (eBioscience) overnight at 4 °C. The following surface antibodies were used: PerCP-Cy5.5 anti-CCR8 (SA214G2; 1:100), APC-Cy7 anti-CD3 (17A2; 1:100), PE-Cy5 anti-CD4 (RM4-5; 1:300), BV421 anti-CD4 (GK1.5; 1:200), FITC anti-CD4 (RM4–5, eBioscience; 1:100), APC anti-CD8α (53–6.7; 1:200), BV510 anti-CD8α (53–6.7; 1:200), BV785 anti-CD8α (53–6.7; 1:200), BV510 anti-CD11b (M1/70; 1:200), PerCP-Cy5.5 anti-CD11b (M1/70; 1:100), BV421 anti-CD11c (N418; 1:200), PE-Cy7 anti-CD11c (N418; 1:250), BV650 anti-CD19 (6D5; 1:150), PE-Cy5 anti-CD24 (M1/69; 1:600), BV510 anti-CD25 (PC61; 1:100), BV785 anti-CD44 (IM7; 1:200), FITC anti-CD45 (30-F11; 1:300), PE/Dazzle 594 anti-CD49b (DX5; 1:100), Alexa Fluor 700 anti-CD62L (MEL-14; 1:150), PE-Cy7 anti-CD68 (FA-11; 1:250), BV605 anti-CD80 (16–10A1; 1:50), PE anti-CD80 (16–10A1; 1:100), PE anti-CD86 (GL-1; 1:100), PE-Cy7 anti-CD103 (2E7; 1:100), PE/Dazzle 594 anti-CTLA-4 (UC10–4B9; 1:100), BV421 anti-F4/80 (BM8; 1:100), BV711 anti-F4/80 (BM8; 1:100), BV421 anti-KLRG1 (2F1/KLRG1; 1:100), BV785 anti-Ly6C (HK1.4; 1:500), PerCP-Cy5.5 anti-Ly6G (1A8; 1:250), Alexa Fluor 700 anti-MHCII (M5/114.15.2; 1:250), BV711 anti-PD-1 (29 F.1A12; 1:100), APC anti-PD-1H (MH5A; 1:100), PE-Cy7 anti-PD-L1 (10 F.9G2; 1:250), PE anti-TCRγδ (UC7–13D5; 1:100), and PE-Cy7 anti-Tim-3 (RMT3–23; 1:100). For intracellular staining, cells were washed with Permeabilization Buffer (eBioscience) following fixation, incubated with directly-conjugated antibodies in Permeabilization Buffer for 30 min on ice, washed, and acquired directly. The following intracellular antibodies were used: PE-Cy5 anti-FoxP3 (FJK-16s, eBioscience; 1:100), PE anti-GATA3 (16E10A23; 1:50), Pacific Blue anti-GranzymeB (GB11; 1:50), PE/Dazzle 594 anti-Helios (22F6; 1:100), PerCP-Cy5.5 anti-IFN-γ (XMG1.2; 1:50), BV421 anti-IL17a (TC11–18H10.1; 1:50), BV650 anti-RORγt (Q31–378, BD Biosciences; 1:50), BV605 anti-T-bet (4B10; 1:50), and BV510 anti-TNF-α (MP6-XT22; 1:50). All antibodies were purchased from BioLegend unless otherwise indicated.

Following fixation, cells were read using 3–11 fluorophore flow cytometry on a LSRFortessa X-20 (BD Biosciences) with 405, 488, 561, and 633 lasers. Compensation matrices were determined for each individual acquisition date using unstained and single fluorophore-stained controls. Data was analyzed using FlowJo software (Tree Star) and cell numbers were calculated from tissue cell counts preceding staining.

### Cell culture and PPP1R13L knockdown

A549 cells (ATCC) were cultured in DMEM (Corning) supplemented with L-glutamine (2 mM), penicillin (100 U/mL), streptomycin (100 μg/mL) and 10% fetal calf serum (FCS; Gibco) at 37 °C in a 5% CO2 humidified atmosphere. *PPP1R13L* knockdown was achieved by cloning a sgRNA targeting the PAM sequence TTGTATGCCCTGGAAGTTGTGG into PX459 (Addgene). A549 cells were transfected with this construct using Lipofectamine (Thermo) following the manufacturer’s protocol and selected with puromycin. Single colonies were expanded and CRISPR/Cas9 indel mutation was validating by sanger sequencing, confirming knockout of the short isoform of iASPP (sviASPP) and knockdown of iASPP.

### ChIP-seq

ChIP-seq of total pol II and p53 were carried out as previously described [130] with 5 µg per IP of anti-pol II antibody (NBP2-32080, Novus Biologicals) and anti-p53 antibodies (9282S, Cell Signaling and sc-126, Santa Cruz). Preparation of ChIP-seq library and ChIP sequencing was prepared with the NEBNext Ultra II DNA Library Prep Kit for Illumina (NEB), according to the manufacturer’s instructions and conducted by the high throughput genomics team of the WTCHG, Oxford. A549 Input (DRR016939), H3K4me1 (DRR016935), and H3K27ac (DRR016938) ChIP-seq were obtained from [131]. Adapters were trimmed with Cutadapt in paired-end mode with the following parameters: -q 15, 10 --minimum-length 10 -A GATCGTCGGACTGTAGAACTCTGAAC -a TGGAATTCTCGGGTGCCAAGG. Obtained sequences were mapped to the human hg19 reference genome with Bowtie2. Properly paired and mapped reads were filtered with SAMtools. PCR duplicates were removed with Picard MarkDuplicates tool. Library-size normalized bedgraph files were created with Bedtools and trackhubs in the UCSC browser were generated with the UCSC bedGraphToBigWig tool. To obtain better peak calling results due to the difficulties of performing ChIP-seq of p53 in A549, for each condition, the ChIP-seq of both p53 antibodies were merged. Peak calling was called from merged biological replicates with MACS2 version 2.1.1.20160309 and the parameters: callpeak –f BAMPE -g 2.9e9 -B -q 0.01 –call-summits. Motif analysis was performed with the MEME Suite. The genomic locations of the AP-1 (JUN/JUNB/JUND/FOS/FOSB/FOSL1/FOSL2), NFκB1/2, and p53 transcription factors were obtained from the Gene Transcription Regulation Database (GTRD) [132]. All the metaclusters were retrieved for each transcription factor and filtered out any metaclusters that did not have support from four independent experiments and from the MACS peak caller. Each genomic location was then overlapped with gene TSS −/+ 10 kb to assign each metacluster to genes.

### mNET-seq

mNET-seq was carried out as previously described [133] with minor changes. In brief, the chromatin fraction was isolated from 1 × 10^7^ A549 cells. Chromatin was digested in 100 µL of MNase (40 units/mL) reaction buffer for 5 min at 37 °C in a thermomixer (1400 rpm). After addition of 10 µL EGTA (25 mM) to inactivate MNase, soluble digested chromatin was collected by 13,000 rpm centrifuge for 5 min. The supernatant was diluted with 400 µL of NET-2 buffer and added to 10 µg of total RNA pol II antibody (MABI0601, MBL International) conjugated with Dynabeads™ M-280 Sheep Anti-Mouse IgG. Immunoprecipitation was performed at 4 °C for 1 h. The beads were washed with 1 ml of NET-2 buffer six times and then with 100 µl of 1xPNKT (1xPNK buffer and 0.05 % Triton X-100) buffer once in the cold room. Washed beads were incubated in 50 µl PNK reaction mix (1xPNKT, 1 mM ATP and 0.05 U/ml T4 PNK 3′phosphatase minus (NEB) in Thermomixer (1400 rpm) at 37 °C for 6 min. After the reaction, the beads were washed with 1 ml of NET-2 buffer once and RNA was extracted with Trizol reagent. RNA was suspended in urea Dye (7 M Urea, 1xTBE, 0.1% BPB and 0.1% XC) and resolved on 6% TBU gel (Invitrogen) at 200 V for 5 min. To size select 30–160 nt RNAs, a gel fragment was cut between BPB and XC dye markers. 0.5 mL tube was prepared with 3–4 small holes made with 25 G needle and placed in a 1.5 mL tube. Gel fragments were placed in the layered tube and broken down by centrifugation at 12,000 *rpm* for 1 min. The small RNAs were eluted from gel using RNA elution buffer (1 M NaOAc and 1 mM EDTA) at 25 °C for 1 h in Thermomixer (900 rpm). Eluted RNA was purified with SpinX column (Coster) with 2 glass filters (Millipore) and the flow-through RNA was ethanol precipitated. RNA libraries were prepared according to manual of NEBNext small RNA library prep kit (NEB). 12–14 cycles of PCR were used to amplify the library. Deep sequencing (Hiseq4000, Illumina) was conducted by the high throughput genomics team of the WTCHG, Oxford.

mNET-seq data were processed as follows: adapters were trimmed with Cutadapt version 1.9.1 [134] in paired-end mode with the following parameters: -q 15, 10 --minimum-length 10 -A GATCGTCGGACTGTAGAACTCTGAAC -a TGGAATTCTCGGGTGCCAAGG. Trimmed reads were mapped to the human hg19 reference sequence with Tophat2 version 2.1.0 [135] and the parameters –g 1 –r 3000 –no-coverage-search. SAMtools version 1.3.1 [136] was used to retain only properly paired and mapped reads (-f 3). A custom python script [137] was used to obtain the 3′ nucleotide of the second read and the strandedness of the first read. Strand-specific bam files were generated with SAMtools. Library-size normalized bedgraph files were created with Bedtools version 2.23 [138] genomecov –bg –scale and trackhubs in the UCSC browser were generated with the UCSC bedGraphToBigWig tool.

### Reads quantification and transcription factors binding sites

Total read base count for mNET-seq data were computed with samtools bedcov tool using strand-specific bam files and normalized to 100 million paired-end reads and to the region’s length. For ChIP-seq, total read base count were computed with samtools bedcov, normalized to 100 million paired-end reads, then the Input signal was subtracted to the IP signal and normalized to the region’s length. Only the regions with a positive signal in at least one sample were kept. For the samples having a signal ≤0 on the remaining regions, their values were put to the minimal value divided by two. The quantification is thus defined for the mNET-seq as:

$$\log _2\frac{{\left[ {{{{\mathrm{region}}}}} \right] \times {{{\mathrm{normalization}}}}\,{{{\mathrm{factor}}}}}}{{{{{\mathrm{length}}}}_{{{{\mathrm{region}}}}}}}$$

and for ChIP-seq as:

$$\log _2\left( {\frac{{\left[ {{{{\mathrm{region}}}}} \right]_{{{{\mathrm{IP}}}}} \times {{{\mathrm{IP}}}}\,{{{\mathrm{normalization}}}}\,{{{\mathrm{factor}}}} - \left[ {{{{\mathrm{region}}}}} \right]_{{{{\mathrm{Input}}}}} \times {{{\mathrm{Input}}}}\,{{{\mathrm{normalization}}}}\,{{{\mathrm{factor}}}}}}{{{{{\mathrm{length}}}}_{{{{\mathrm{region}}}}}}}} \right)$$

The quantification regions is defined as follow: Gene body (GB) of protein-coding genes: TSS + 0.5 kb to TES. The lists of transcription factors binding sites associated with down-regulated or up-regulated genes were obtained with the DAVID Bioinformatics Resources [139, 140].

### Gene set enrichment analysis

Gene set enrichment analysis was performed using fgsea (version 3.15). Genes were ranked according to log2FC and used to query GOBP signature libraries. Network plot was generated using clusterProfiler (version 1.0.12).

### RNA-seq and principal component analysis

RNA was prepared from snap-frozen tissues by grinding with mortar and pestle in liquid nitrogen, followed by tissue homogenization in 600 μL Buffer RLT (Qiagen) with β-mercaptoethanol and RNA isolation using the RNeasy Mini Kit according to manufacturer’s protocols. Following mRNA isolation, libraries were prepared using a QuantSeq 3′ mRNA-seq Library Kit (Lexogen) and sequenced on a HiSeq 4000 (Illumina). FASTQ files were aligned using BWA-mem to the GRCm38.ERCC genome, counted using featureCounts, and annotated using the TxDb.Mmusculus.UCSC.mm10.knownGene database. Normalized counts were obtained using the VST normalization function of DeSeq v1.16.1 and used to generate expression scatter plots and volcano plots. Principal component analysis was performed using plotPCA. All RNA-Seq analyses were conducted in RStudio.

### Statistical analyses

Statistical analysis was performed using unpaired two-tailed Student’s *t* tests where indicated. Where multiple comparisons were performed, statistical significance was determined using the two-stage linear step-up procedure of Benjamini, Krieger and Yekutieli with a false-discovery rate (Q) = 5% without assuming consistent standard deviations [141]. EAE clinical severity was analyzed using non-compartmental estimation of the area under the curve (AUC) using the log-trapezoidal rule. Median EAE duration and T_reg_ infiltration was compared using the Mann–Whitney *U* test due to non-normal distribution. Disease-free survival and overall survival Kaplan–Meier estimations were compared using the log-rank Mantel-Cox test with one degree of freedom, and hazard ratios were calculated using the Mantel–Haenszel method [142]. MOG_35-55_ stimulation-induced T cell proliferation was analyzed using two-way ANOVA with between-subject factors of genotype and within-subject factors of peptide concentration. Where Student’s *t* tests and ANOVA were performed, normality was tested using the D’Agostino and Pearson test with *p* = 0.05. Variance was compared using an *F* test for unpaired *t*-tests and the Bartlett’s test for ANOVA. Fisher’s exact test was performed on contingency tables to determine statistical significance of differences in tumor grade between different genotypes. *P* values <0.05 were considered significant, except in cases where adjusted *q* values <0.05 were used to account for false discovery. For calculations of total cell counts, outliers were removed using robust nonlinear regression [143] with Q = 1%. All analyses were performed in Prism 7 (GraphPad Software). Data are presented as mean ± standard error of the mean (s.e.m.).

## Supplementary information

Supplemental Figure Legends

Nature Checklist

Supplemental Figure S1

Supplemental Figure S2

Supplemental Figure S3

Supplemental Figure S4

Supplemental Figure S5

Supplemental Figure S6

Supplemental Figure S7

## Data Availability

All ChIP-seq and mNET-seq data generated in this study have been deposited in the GEO database and are available under primary accession number GSE202445. RNA-seq data generated in this study have been deposited in the GEO database and are available under primary accession number GSE218389.
