# Supplementary material for: Regulation of immunological tolerance by the p53-inhibitor iASPP
Source: Cell Death Dis. 2023 Feb 6;14(2):84. doi: 10.1038/s41419-023-05567-9 (PMC9902554; doi:10.1038/s41419-023-05567-9)
Supplement: Supplementary file 1 — Supplemental Figure Legends [file 41419_2023_5567_MOESM1_ESM.docx]

**Supplemental Figure Legends**

**Figure S1**. iASPP Deficient Mice Have Altered CD4^+^ T Cell Phenotypes

(A) Flow cytometric quantification of CD3^+^CD4^+^FoxP3^+^ T_reg_ cell total frequency from indicated organs of WT or *iASPP^-/-^* mice.

(B) Representative flow cytometry contour plots of gating strategy used to identify CD3^+^CD4^+^FoxP3^+^ T_reg_ cells.

(C) Flow cytometric quantification of CD3^+^CD4^+^FoxP3^+^ T_reg_ cell frequency from indicated organs of WT or *iASPP^-/-^* mice.

(D) Flow cytometric quantification of PD-1 expression by CD4^+^ T cells from indicated organs of WT or *iASPP^-/-^* mice.

(E) Flow cytometry histograms (left) and quantification (right) of PD-1H expression by CD4^+^ or γδ T cells from livers, spleens, or thymuses WT or *iASPP^-/-^* mice.

(F) Schematic (left) and flow cytometry contour plots (right) of WT or *iASPP^-/-^* naive CD4^+^ T cell (T_naive_) differentiation under Th1, Th2, Th17, or T_reg_ polarizing conditions.

**Figure S2.** iASPP Deficiency Attenuates CD4 Responses in Experimental Autoimmune Encephalitis

(A) Kaplan-Meier estimate of disease-free survival. Survival was tracked until 30 days after immunization.

(B) Column scatter plot showing days WT or *iASPP^-/-^* mice with EAE spent above a clinical severity score of 1.

(C) Quantification of demyelination in indicated brain regions in WT or *iASPP^-/-^* mice 30 days after immunization with MOG/CFA. Each point represents mean LFB intensity in a single field of view.

(D) Quantification of CD3^+^ T cells in indicated brain regions in WT (*n =* 4) or *iASPP^-/-^* (*n =* 6) mice 30 days after immunization with MOG/CFA. Each point represents the average CD3 infiltration across three or more fields of view for a single mouse.

(E) Cell numbers in brains of WT (*n* = 5) or *iASPP^-/-^* (*n* = 7) mice with EAE determined by flow cytometry.

(F) Flow cytometry analysis of EAE splenocytes stimulated with MOG and IL-2 for 72 hr analyzed by CFSE dilution. Data are mean ± s.e.m.

(G) Flow cytometric quantification of PD-1H expression in splenocytes from (E).

**Figure S3.** Pancreatic Tumors Lacking iASPP Have an Exhausted T cell Response

(A) CD4 (top) and CD8 (bottom) stains of pancreata isolated from *Kras^LSL-G12D/+^;Pdx1-Cre* (KC, *n =* 5), *Kras^LSL-G12D/+^;iASPP^fl/fl^;Pdx1-Cre* (KC;iASPP^Δ8/Δ8^, *n =* 5), or *Kras^LSL-G12D/+^;Trp53^LSL-R172H/+^;Pdx1-Cre* (KPC, *n =* 5) mice. Scale, 100 μm. ADM, acinar to ductal metaplasia; PanIN, pancreatic intraepithelial neoplasia; PDAC, pancreatic ductal adenocarcinoma.

(B) Column scatter plot shows quantification of pancreatic neoplasia-infiltrating CD4 (left) and CD8 (right) cells per area.

(C) Flow cytometry histograms (left) and quantification (right) of PD-1 and Tim-3 expression in CD4 and CD8 cells from KC (blue) or KC;iASPP^Δ8/Δ8^ (red) pancreata.

**Figure S4.** Myeloid cells are Activated in Pancreatic Tumors Lacking iASPP

(A) Flow cytometry histograms of CD80, PD-1, PD-1H, and MHC II expression in pancreata-infiltrating MDSC-M, MDSC-P, DC, and macrophages from KC (blue) or KC;iASPP^Δ8/Δ8^ (red) pancreata.

(B) Quantification of CD80, PD-1, PD-1H, and MHC II expression in pancreata-infiltrating MDSC-M, MDSC-P, DC, and macrophages from KC (blue) or KC;iASPP^Δ8/Δ8^ (red) pancreata.

**Figure S5.** iASPP Deficiency Promotes CD4+ T Cell PD-1 Expression in Lung Cancer

(A) HE, CD3, and FoxP3 immunohistochemistry stains in lung lobes 15 weeks after tumor initiation. Scale, 100 μm.

(B) Flow cytometric quantification of PD-1 expression by indicated cells from mediastinal lymph nodes (mLN) and lungs of K (blue) or Ki (red) mice 15 weeks after tumor initiation.

**Figure S6.** iASPP Regulates Immune and Apoptosis Related Genes

(A) Principal component analysis from VST normalized gene counts from RNA-seq of iC (*n* = 3), KC (*n* = 7), KC;iASPP^Δ8/Δ8^ (*n* = 8), or KPC (*n* = 4) pancreatic cancer lysates.

(B) Average VST normalized gene counts from RNA-seq analysis of murine pancreatic cancer lysates.

(C) Gene browser visualization of RNA pol II occupancy assessed by mNET-seq in the *Vsir* gene from wild type or iASPP (*PPP1R13L*) knockdown A549 cells. H3K4me1 and H3K27ac active transcription marks in wild type A549 cells are shown below for reference.

(D) Gene set enrichment plot of indicated gene module against genes ranked by fold enrichment in iASPP (*PPP1R13L*) knockdown A549 cells measured by total pol II mNET-seq.

**Figure S7.** iASPP in the Balance Between Tolerance and Autoimmunity

Hypothetical model of the role iASPP plays in promoted non-tolerogenic cell death. The immunological consequences of phagocytosis and antigen presentation are determined by the underlying cause of cell death. Infection or necrosis and corresponding secretion of pro-inflammatory cytokines induces co-stimulation by antigen-presenting cells (APCs). Engagement of T cell receptor (TCR) with co- stimulation and pro-inflammatory cytokine release stimulates T cell activation, tipping the immunological balance towards immunity. During normal tissue turnover, p53- induced apoptosis and phagocytosis stimulates secretion of anti-inflammatory cytokines and antigen presentation without co-stimulation. Anergic TCR engagement and expression of checkpoint molecules tips the immunological balance towards tolerance. Treg, regulatory T cell.
