# Supplementary figures and images for: Regulation of immunological tolerance by the p53-inhibitor iASPP
Source: Cell Death Dis. 2023 Feb 6;14(2):84. doi: 10.1038/s41419-023-05567-9 (PMC9902554; doi:10.1038/s41419-023-05567-9)

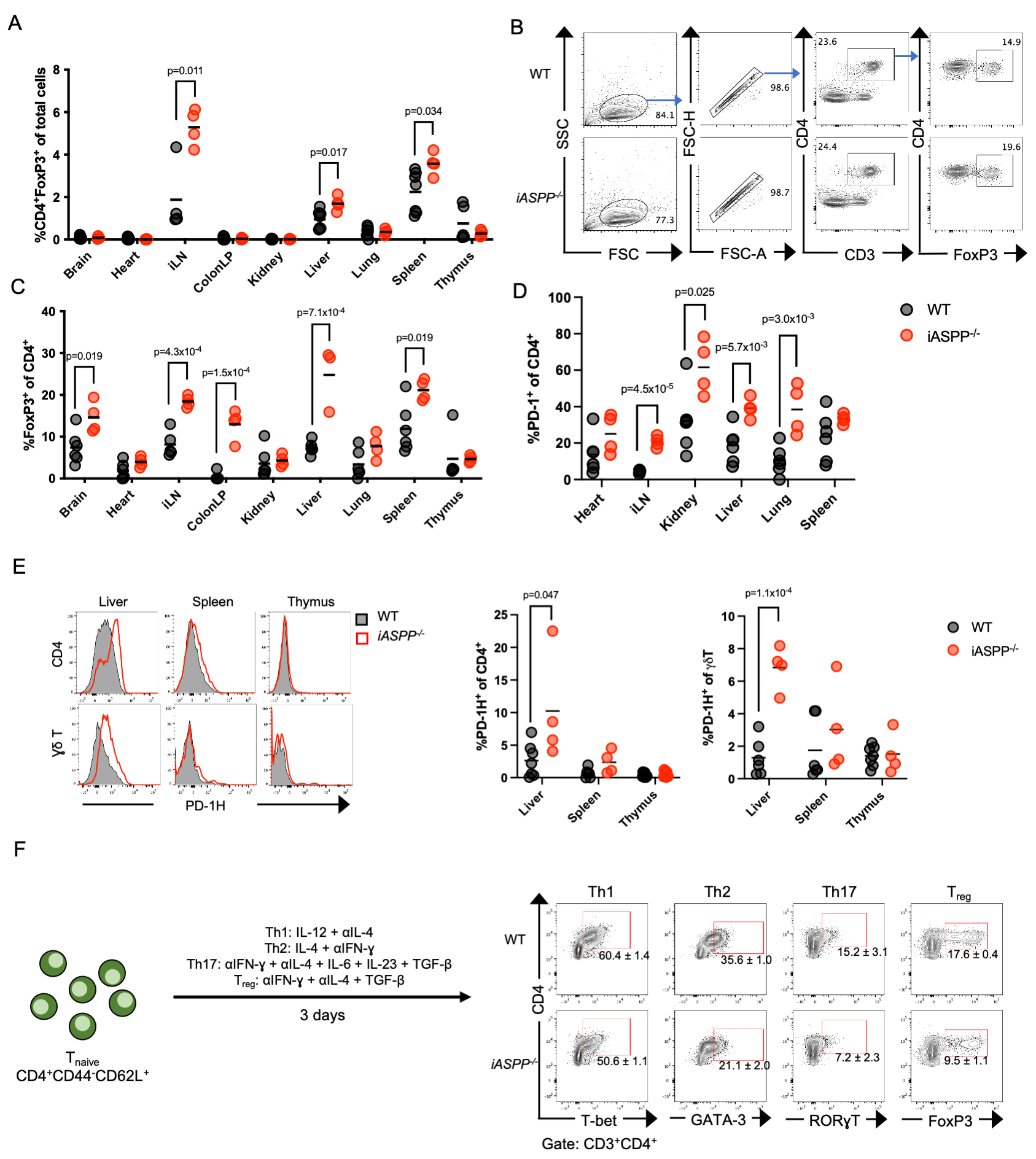

Supplement: Supplementary file 3 — Supplemental Figure S1 [file 41419_2023_5567_MOESM3_ESM.tif]

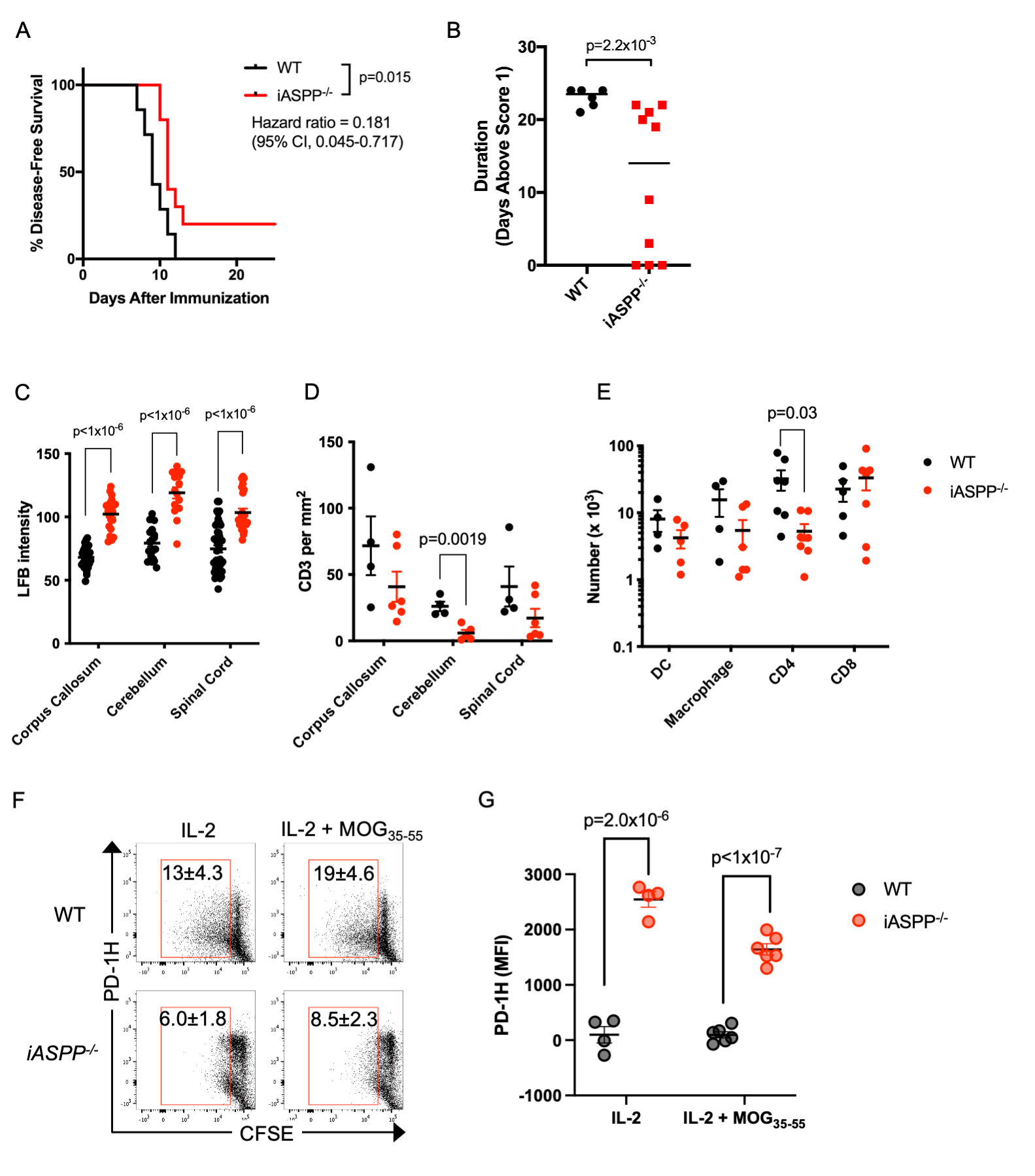

Supplement: Supplementary file 4 — Supplemental Figure S2 [file 41419_2023_5567_MOESM4_ESM.tif]

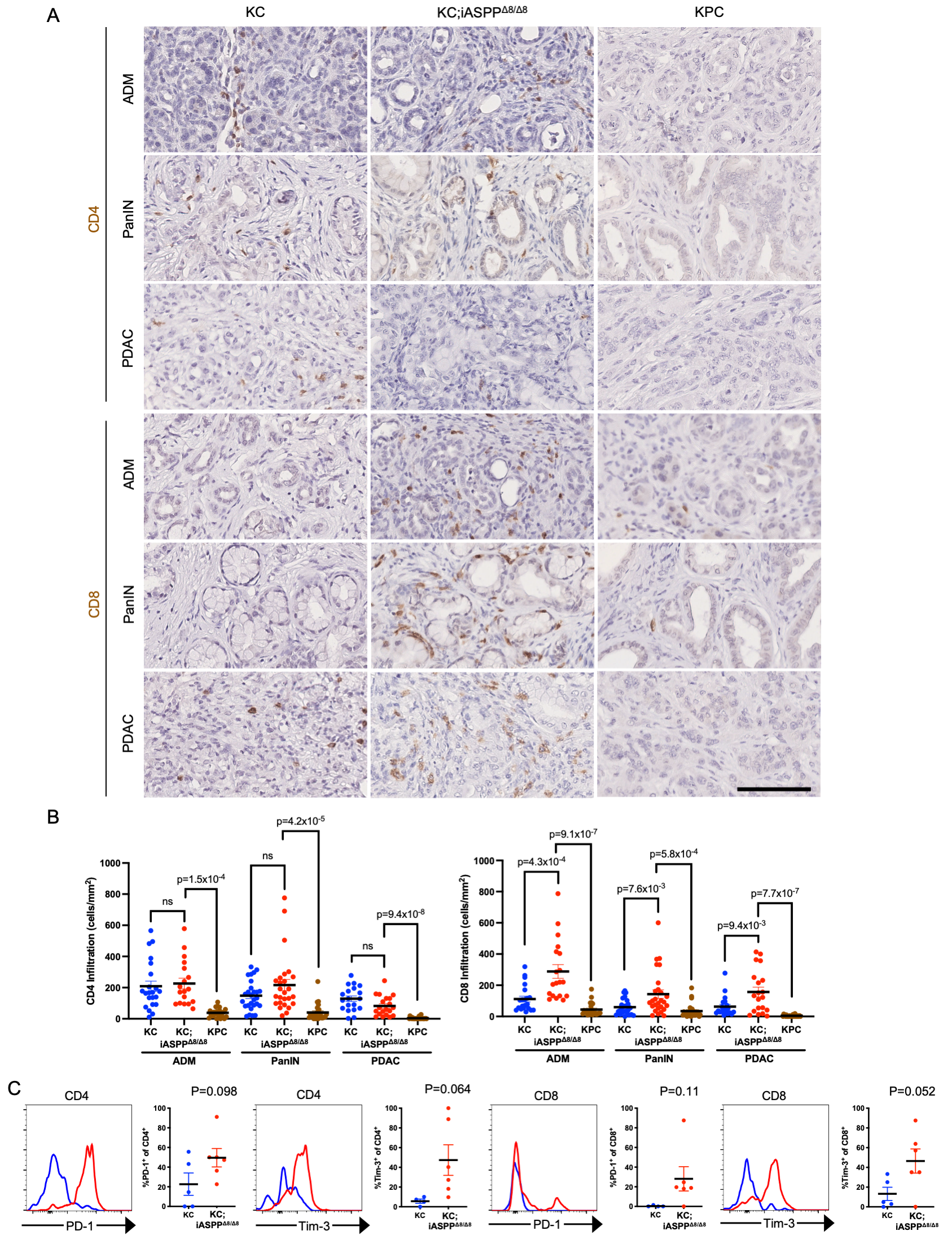

Supplement: Supplementary file 5 — Supplemental Figure S3 [file 41419_2023_5567_MOESM5_ESM.tif]

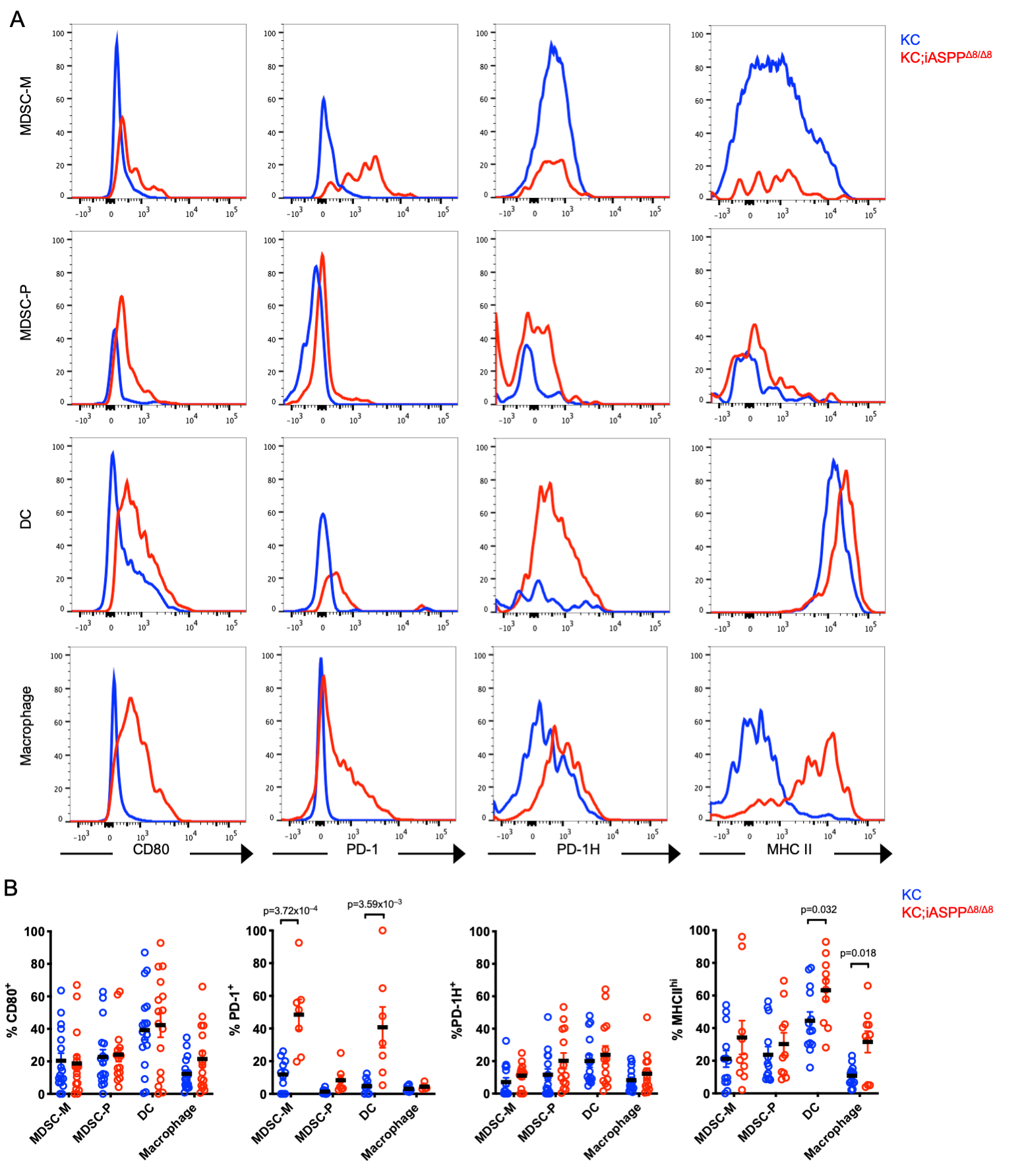

Supplement: Supplementary file 6 — Supplemental Figure S4 [file 41419_2023_5567_MOESM6_ESM.tif]

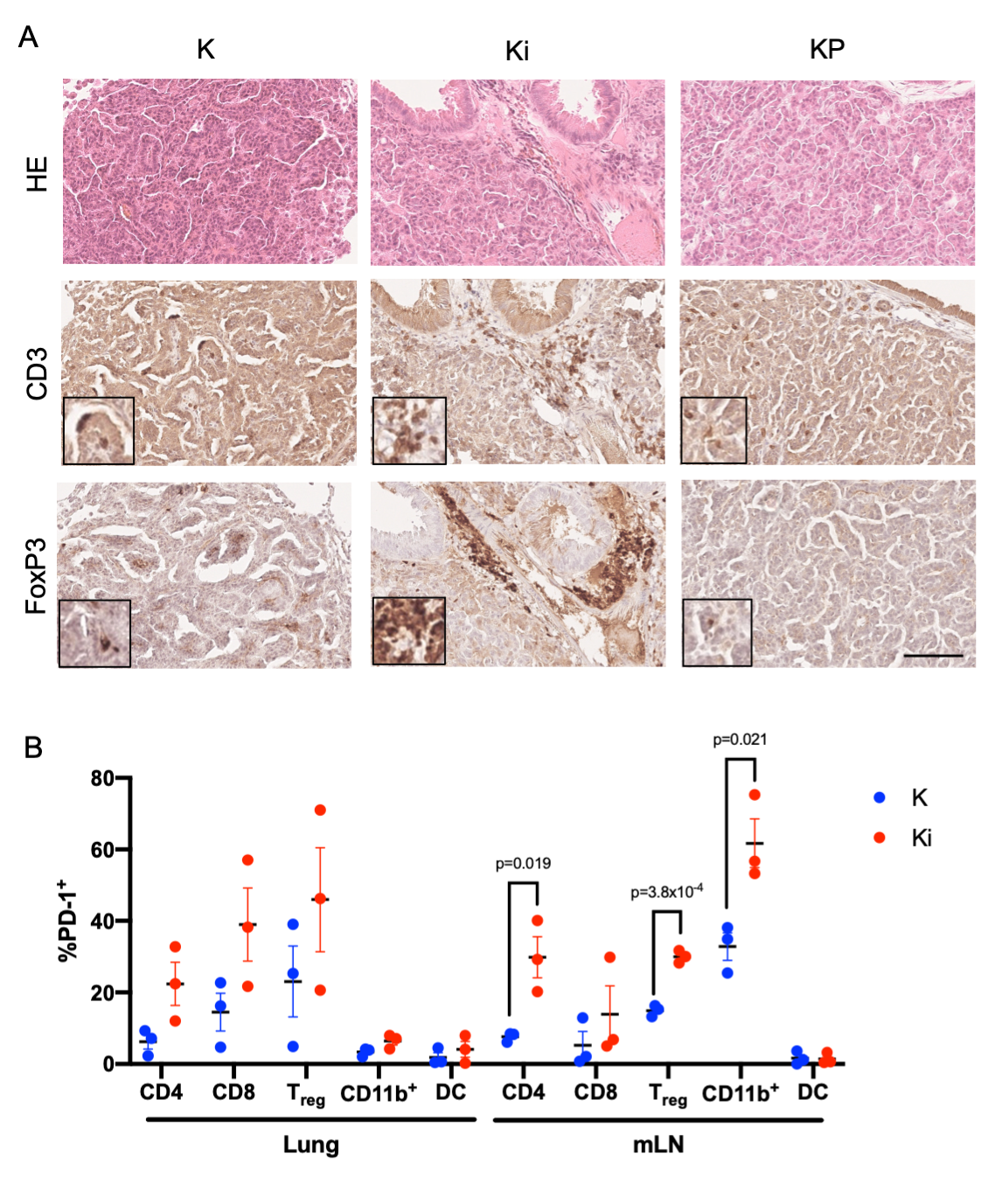

Supplement: Supplementary file 7 — Supplemental Figure S5 [file 41419_2023_5567_MOESM7_ESM.tif]

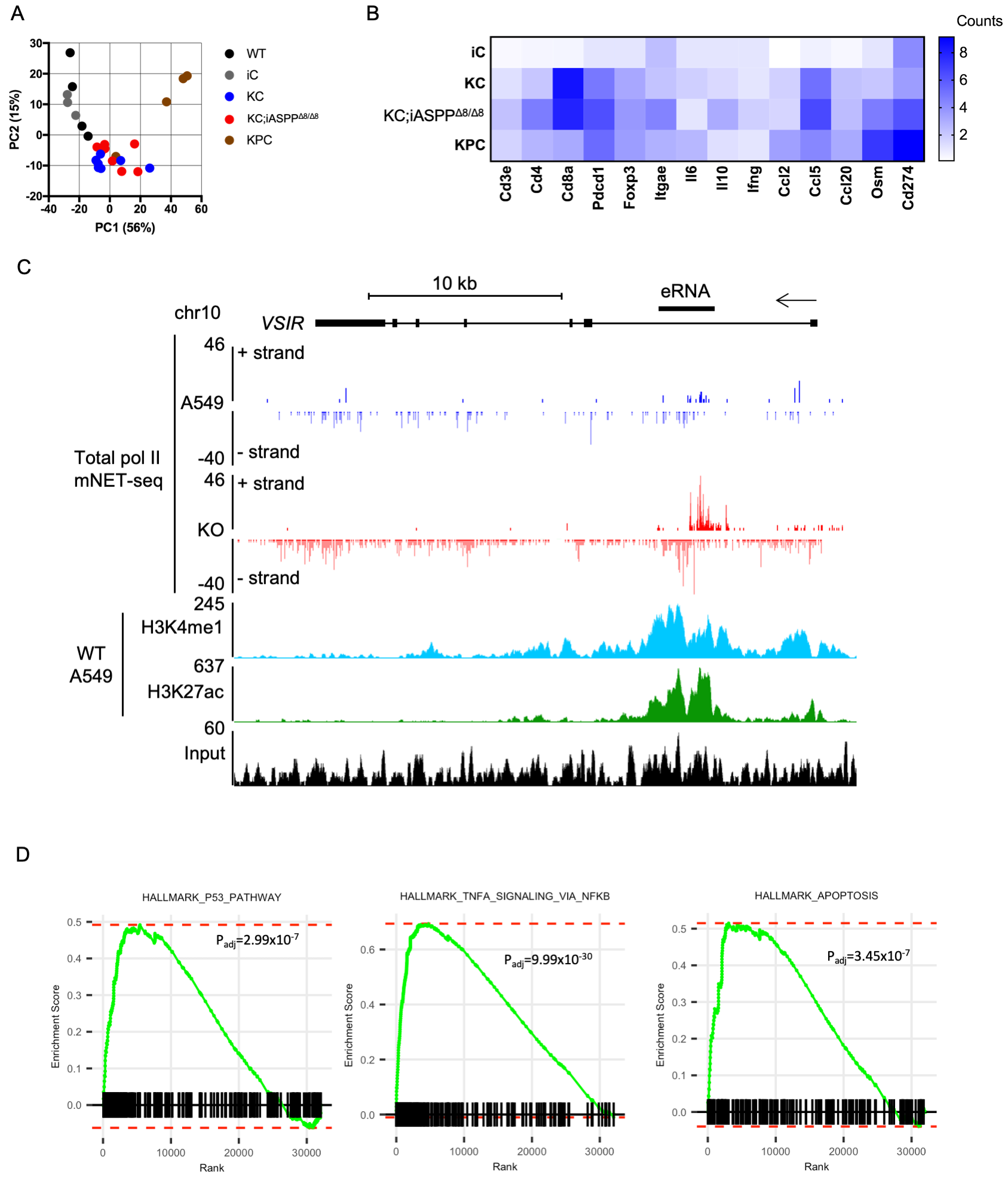

Supplement: Supplementary file 8 — Supplemental Figure S6 [file 41419_2023_5567_MOESM8_ESM.tif]

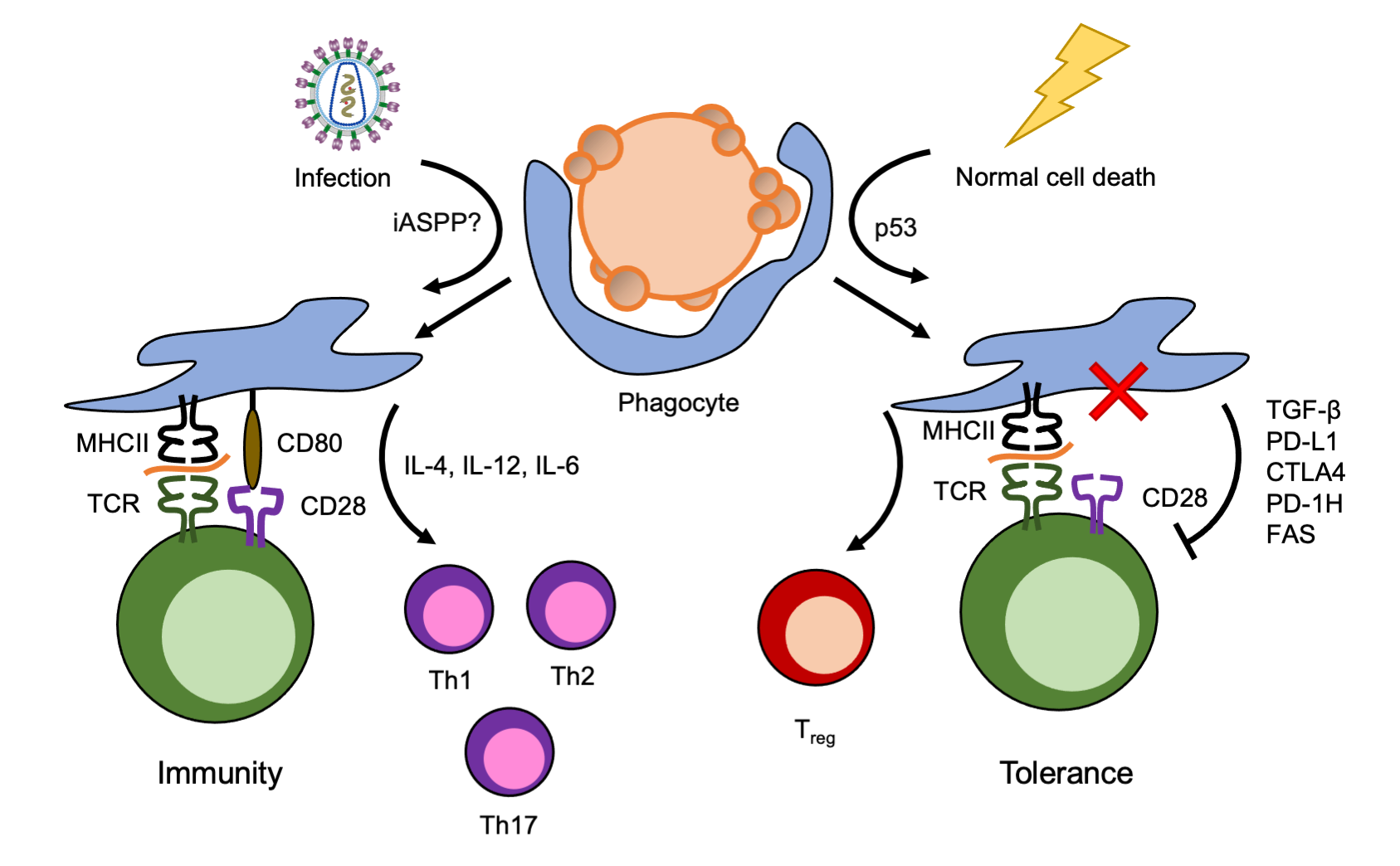

Supplement: Supplementary file 9 — Supplemental Figure S7 [file 41419_2023_5567_MOESM9_ESM.tif]
